# Reconstruction of full-length LINE-1 progenitors from ancestral genomes

**DOI:** 10.1093/genetics/iyac074

**Published:** 2022-05-12

**Authors:** Laura F Campitelli, Isaac Yellan, Mihai Albu, Marjan Barazandeh, Zain M Patel, Mathieu Blanchette, Timothy R Hughes

**Affiliations:** Department of Molecular Genetics, University of Toronto, Toronto, ON M5S 1A1, Canada; Donnelly Centre, University of Toronto, Toronto, ON M5S 1A1, Canada; Department of Molecular Genetics, University of Toronto, Toronto, ON M5S 1A1, Canada; Donnelly Centre, University of Toronto, Toronto, ON M5S 1A1, Canada; Donnelly Centre, University of Toronto, Toronto, ON M5S 1A1, Canada; Donnelly Centre, University of Toronto, Toronto, ON M5S 1A1, Canada; Faculty of Pharmaceutical Sciences, University of British Columbia, Vancouver, BC V6T 1Z4, Canada; Department of Molecular Genetics, University of Toronto, Toronto, ON M5S 1A1, Canada; Donnelly Centre, University of Toronto, Toronto, ON M5S 1A1, Canada; Department of Computer Science, McGill University, Montreal, QC H3A 0G4, Canada; Department of Molecular Genetics, University of Toronto, Toronto, ON M5S 1A1, Canada; Donnelly Centre, University of Toronto, Toronto, ON M5S 1A1, Canada

**Keywords:** ancestral sequence reconstruction, evolutionary arms race, KRAB zinc finger protein, LINE-1

## Abstract

Sequences derived from the Long INterspersed Element-1 (L1) family of retrotransposons occupy at least 17% of the human genome, with 67 distinct subfamilies representing successive waves of expansion and extinction in mammalian lineages. L1s contribute extensively to gene regulation, but their molecular history is difficult to trace, because most are present only as truncated and highly mutated fossils. Consequently, L1 entries in current databases of repeat sequences are composed mainly of short diagnostic subsequences, rather than full functional progenitor sequences for each subfamily. Here, we have coupled 2 levels of sequence reconstruction (at the level of whole genomes and L1 subfamilies) to reconstruct progenitor sequences for all human L1 subfamilies that are more functionally and phylogenetically plausible than existing models. Most of the reconstructed sequences are at or near the canonical length of L1s and encode uninterrupted ORFs with expected protein domains. We also show that the presence or absence of binding sites for KRAB-C2H2 Zinc Finger Proteins, even in ancient-reconstructed progenitor L1s, mirrors binding observed in human ChIP-exo experiments, thus extending the arms race and domestication model. RepeatMasker searches of the modern human genome suggest that the new models may be able to assign subfamily resolution identities to previously ambiguous L1 instances. The reconstructed L1 sequences will be useful for genome annotation and functional study of both L1 evolution and L1 contributions to host regulatory networks.

## Teaser

To elucidate the molecular history of mammalian LINE-1 elements, Campitelli, Yellan *et al*. reconstruct functionally and phylogenetically plausible ancestral sequences for dozens of LINE-1 subfamilies. These reconstructions empower investigation into how transcription factor binding to modern fossilized LINE-1s may have arisen through an arms race with their active ancestors. The new models may be able to improve LINE-1 annotations in the human genome; those reconstructed LINE-1 sequences will be useful for functional studies of LINE-1 evolution and contributions to host regulatory networks.

## Introduction

Endogenous retroelements (EREs) are copy-and-paste transposons that have expanded to occupy at least half of the human genome. ERE insertions can be detrimental, but they have also been co-opted as myriad functional elements (Feschotte 2008; Chuong *et al.* 2017). Some ERE classes contain strong transcriptional activating sequences (de Souza *et al.* 2013; Jacques *et al.* 2013; Miao *et al.* 2020), and have given rise to numerous lineage-specific host *cis*-regulatory elements (Villar *et al.* 2015; Cao *et al.* 2019), including many affecting embryonic development (Kunarso *et al.* 2010).

The LINE-1 (L1) (Long INterspersed Element) family is a dominant class of EREs in mammals, collectively occupying ∼17% of the human genome (Lander *et al.* 2001). Human L1s are grouped into ∼67 subfamilies, which are defined as closely related groups presumably originating from one or a few closely related progenitor sequences (Smit *et al.* 1995; Khan *et al.* 2006). The L1 subfamilies represent a nearly continuous vertically transmitted lineage that originated near the base of eutherian mammals (>160 million years old) (Burton *et al.* 1986). The human-specific L1HS subfamily represents the only currently active subfamily in the human genome; the remainder are present only as fossils.

**Fig. 1.**
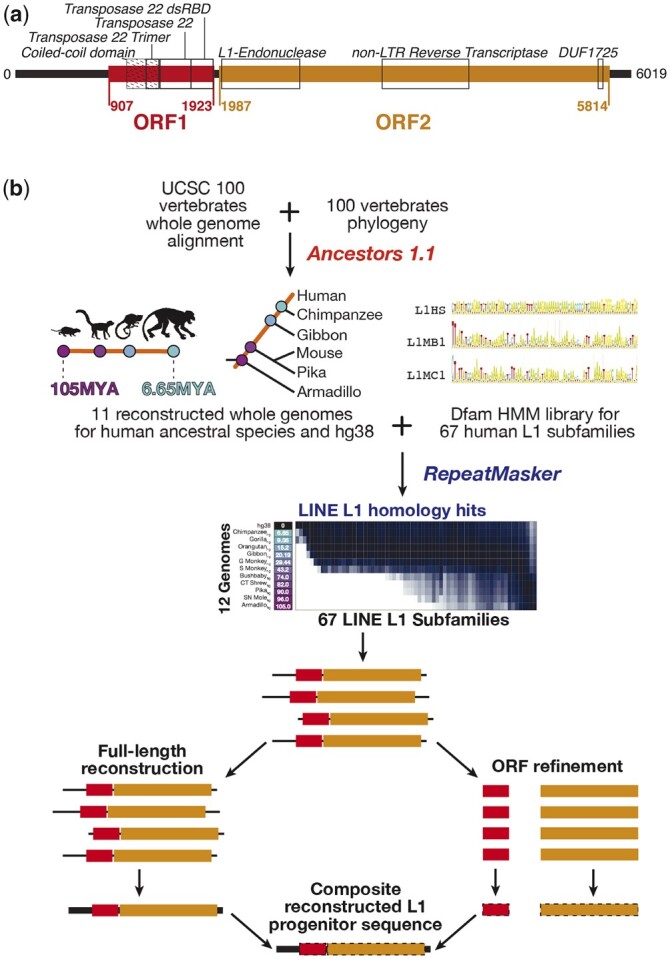
Overview of L1 elements and methodology. a) Schematic of the (Khan *et al.* 2006) L1HS subfamily model, showing the locations of the ORFs and domains. Domains are labeled with black boxes, and according to their CDD names (note that these names are misnomers relative to current understanding of LINE-1s). In ORF1, “Transposase 22 dsRBD” is the CTD, “Transposase 22” is the RRM, and “Transposase 22 Trimer” the N-terminal section of the coiled-coil domain. The full length of the coiled-coil domain is indicated with diagonal hatching. b) Workflow used to annotate L1 loci in ancestral genomes and reconstruct L1 progenitors. The sequence logos represent segments of Dfam L1 profile HMMs.

Functional L1s are 6–7 kb in length and are expressed as a single transcript that encodes 2 protein-coding ORFs (ORF1 and ORF2) and also contains a long 5′ end with RNA polymerase II promoter activity (Swergold 1990) (Fig. 1a). The internal promoters of active mouse and human L1s are most active in the germline and early embryo, as well as during neural development and in cancer (Faulkner and Garcia-Perez 2017). The ORF protein products enable autonomous retrotransposition of L1s (Moran *et al.* 1996): ORF1p contains a coiled-coil domain, a single-stranded RNA binding noncanonical RRM, and a C-terminal domain (CTD) that assists RRM binding (Khazina *et al.* 2011) (Fig. 1a). ORF1 directs L1 RNP assembly (Hohjoh and Singer 1996; Kolosha and Martin 1997), and possesses nucleic acid chaperone activity integral for retrotransposition (Martin *et al.* 2005). ORF2 contains apurinic/apyrimidinic endonuclease (APE) and reverse transcriptase (RT) domains, which facilitate integration site cleavage and reverse transcription of L1 RNA, respectively (Mathias *et al.* 1991; Feng *et al.* 1996). As the L1 RNA is bicistronic, ORF2 translation may be facilitated by a predicted Internal Ribosomal Entry Site (IRES) located in the inter-genic region (IGR) of some mammalian L1s (Boissinot and Sookdeo 2016). However, the very short IGR of the youngest human L1, L1HS (also called L1PA1) does not contain an IRES, and its ORF2 may instead be translated via a poorly characterized ribosome reinitiation mechanism (Alisch *et al.* 2006). A 5′ antisense ORF0 peptide with no known domains is also encoded by primate-specific L1s (L1HS-L1PA8) and may function to enhance retrotransposition activity (Denli *et al.* 2015).

EREs can drive the evolution of host mechanisms that suppress transposition. A striking example is the “arms race” between EREs and mammalian KRAB-C2H2 zinc finger repressors (KZFPs) (Castro-Diaz *et al.* 2014). KZFPs are the largest and most rapidly evolving family of human transcription factors (Lambert *et al.* 2018), presumably due to their predominant role in binding and repressing EREs. The molecular evolutionary histories of EREs in turn reflect signatures of past selection imposed by transposition-suppressing factors (Castro-Diaz *et al.* 2014), including KZFPs. In one well-documented case, ZNF93 was found to bind and suppress L1 subfamilies, apparently driving selection for later L1 subfamilies to acquire a deletion of the ZNF93-binding site (Jacobs *et al.* 2014). A subsequent analysis of the genomic binding sites of 222 human KZFPs found that many KZFPs that bind Endogenous Retroviruses and LINEs arose at about the same time as their transposon targets, and identified mutation events in young (primate-specific) L1s that correlated with loss of KZFP binding for 4 KZFPs (including ZNF93) (Imbeault *et al.* 2017). Importantly, the ERE fossils, as well as the proteins that evolve to bind and silence them, can also contribute to host regulatory processes, a phenomenon referred to as “domestication” (Kunarso *et al.* 2010; Bruno *et al.* 2019).

Despite their prevalence and importance, the evolutionary trajectories of L1s and their interactions with host factors are relatively difficult to trace, partly because extant copies are degraded due to their age, and also because they are predominantly truncated. L1 transposition is particularly error-prone, often producing an insertion that is severely truncated at the 5′ end (Lander *et al.* 2001) because of premature interruption of L1 reverse transcription by nonhomologous end joining factors (Gilbert *et al.* 2005; Suzuki *et al.* 2009; Coufal *et al.* 2011). Each distinct L1 subfamily is currently defined by consensus sequences [housed in Repbase (Bao *et al.* 2015)] and corresponding Hidden Markov Models (HMMs) [housed in Dfam (Hubley *et al.* 2016)], which are based on ∼900 bp of the 3′ end. Supplemental sequences and HMMs represent generalized components of L1 5′ ends and ORFs that do not map one-to-one to the 67 L1 subfamilies (Smit *et al.* 1995; Bao *et al.* 2015; Hubley *et al.* 2016). These subsequence models are used by RepeatMasker, which has been used to produce commonly referenced human genome annotations on UCSC Genome Browser. But, full-length progenitor sequences cannot be generated by simply combining existing L1 subsequence models, since the generalized components are not subfamily-specific. Previous work has derived full-length progenitor sequences for the 22 most recent L1 subfamilies, which are primate-distributed, by approximating the ancestral sequence to their longest insertions in the human genome (Scott *et al.* 1987; Boissinot and Furano 2001; Khan *et al.* 2006). In these sequences, the 5′ ends vary greatly between subfamilies; this variability apparently corresponds with KZFP binding escape in some cases (Jacobs *et al.* 2014; Imbeault *et al.* 2017). Reconstruction of progenitor sequences for human L1 subfamilies distributed beyond primates has not been reported, presumably because of complications introduced by the greater degradation of older L1 instances in the modern human genome.

Here, we reconstructed full-length primate and mammalian-distributed L1 subfamily progenitor sequences from reconstructed ancestral mammalian genomes, which should contain L1 copies that more closely resemble the progenitors (Blanchette *et al.* 2004). Sequence reconstructions employ sequence alignments and their phylogeny in order to infer an ancestral sequence (Blanchette *et al.* 2008). Figure 1b gives an overview of the approach taken. We first used the UCSC 100-way vertebrate alignment (Miller *et al.* 2007) to produce 11 reconstructed ancestral human genomes, using Ancestors 1.1 (Diallo *et al.* 2010) (Fig. 1b, top; Supplementary Fig. 1) (the most distant being the LCA with armadillo, representing the eutherian common ancestor). We then annotated these genomes with RepeatMasker (Fig. 1b, middle), and compiled the longest matches to each of the L1 subfamilies within each genome. We then generated an alignment, phylogeny, and maximum-likelihood ancestral reconstructed sequence of the putative common progenitor for each subfamily in each genome, first using standard nucleotide sequence alignment, then refining the ORFs using a codon-aware aligner (Fig. 1b, bottom). We evaluated the reconstructed L1s using multiple criteria, including length, identity to standards, presence of protein domains, and comparison to their established phylogeny, in order to produce a new set of reference L1s in which most of the entries are nearly complete according to these criteria. Finally, we illustrate the utility of the reconstructed sequences by examining how motif matches for KZFPs correspond to observed ChIP binding to L1s, and by using them to reannotate the human genome.

## Materials and methods

### Ancestral reconstructed genomes

We obtained reconstructed ancestral genomes using Ancestors 1.1 (Diallo *et al.* 2010), using the mammalian clade of UCSC’s 100-way whole genome alignment and the corresponding UCSC phylogeny. The 11 ancestral genomes on the human lineage were extracted, ranging from the human-armadillo common ancestor (105 MYA) to the human-chimpanzee common ancestor (7 MYA). hg38 was added to this dataset for a total of 12 genomes.

We extracted Progressive Cactus ancestral genomes from the 200-mammal whole-genome alignment (Armstrong *et al.* 2020), which are generated as part of the program’s alignment strategy. The selected genomes correspond to the common ancestors of all eutherians, primates, and simians in the alignment’s species tree (nodes fullTreeAnc239, fullTreeAnc110, and fullTreeAnc110point5, respectively). The corresponding Ancestors 1.1 genomes are Armadillo_hg_, Squirrel Monkey_hg_, and Bushbaby_hg_.

### Initial L1 annotations

We annotated L1s and all other repetitive genomic elements in each of the 12 Ancestors genomes using RepeatMasker, version open-4.0.7 (Tempel 2012) in sensitive mode, choosing the nhmmscan v3.1b2 (February 2015) algorithm, and Dfam 2.0 HMMs (Hubley *et al.* 2016) serving as the reference library for repeat element classification. We excluded loci annotated by RepeatMasker as belonging to generalized 5′ end subfamilies (presumably because the 3′ end of the locus cannot be confidently mapped to a specific 3′ end subfamily).

For each of the 67 L1 subfamilies defined by Dfam 3′ end HMM models, we extracted the most probable linear sequences from the HMMs using HMMER hmmemit -c (Finn *et al.* 2011). Then, for each subfamily in each genome, we calculated the average sequence divergence between the subfamily’s 3′ end HMM hit loci and the 3′ end HMM consensus model, using RepeatMasker’s calcDivergenceFromAlign.pl. This method calculates the sequence divergence based on the Kimura 2 Parameter substitution model, and applies CpG correction to account for the hypermutability of CpG sites (i.e. 5′-CG-3′ sites, where C–G base pairs are frequently converted to A–T due to cytosine methylation and spontaneous deamination).

### Full-length progenitor sequence reconstruction

For each of the 67 L1 subfamilies, we recovered the sequences of the 100 longest hits from each of the 12 genomes (hg38 and the 11 ancestors), excluding any hits <3 kb in length. For each genome and subfamily combination, we produced a multiple sequence alignment of these ≤100 longest hits, using Muscle (Edgar 2004). We then supplied each alignment to FastML v3.11 (Ashkenazy *et al.* 2012) to reconstruct the ancestral sequence to all sequences in the alignment, using the generalized time reversible (GTR) evolutionary model instead of the default JC for ancestral state inference, which improved recovery of less-conserved (and often truncated) 5′ ends. FastML by default returned 2 possible solutions for the ancestral sequence of the sequences in the input multiple sequence alignment, using either a maximum likelihood or parsimony-based algorithm for indel modeling. The result of this process was a maximum of 24 candidate progenitor sequences (a maximum of 2 for each genome) for each of the 67 subfamilies. Given that high-quality hits of all subfamilies are not present in all genomes, the total number of candidate progenitor sequences reconstructed was 1,134.

### Automated selection of the best candidate progenitor sequence

We selected a single “best” progenitor sequence for each subfamily based on length and identity to gold standard sequences (up to 3 per subfamily, as described in the *Results*) (Khan *et al.* 2006; Bao *et al.* 2015; Hubley *et al.* 2016). Identity between the 2 sequences was expressed as a percentage, taken as (a− b)/a, where a is the length of the shorter sequence (either the candidate progenitor or the gold standard) and b is the total number of mismatches in the pairwise local alignment between the sequences. Indels were not penalized because the selection pipeline includes a separate length criterion, and because most gold standard sequences were truncated.

For each subfamily, the maximum % identity among all candidate reconstructed sequences and all gold standards was determined. The best full-length reconstructed sequence was then selected by first removing candidates with no % identities to any gold standard within 1.5% of this maximum value. Reconstructed sequences with lengths >8 kb were also discarded, to avoid inclusion of insertions in cases where there were few sequences in the initial alignment. Candidate progenitors for each subfamily were then rank ordered by highest % identity to a gold standard, followed by highest normalized length (with all lengths between 6 and 8 kb considered equally preferrable), and the top-ranking candidate selected as the best full-length progenitor sequence.

### Reconstruction of progressive cactus-derived L1 progenitor sequences

To facilitate comparison to the Ancestors 1.1 ancestral genome derived L1 reconstructed sequences, we scanned the Cactus genomes with RepeatMasker, using the same library and parameters as those applied to the Ancestors genomes. We generated the full-length reconstructed sequences using the same hit filtering criteria, alignment method, and FastML parameters (see sections above). The Cactus L1MA2, L1MA4, and L1MD1 were reconstructed using hits derived from the Cactus ancestral simian genome, while L1MD2 and L1MD3 were reconstructed using hits from the Cactus primate genome, for comparability to those derived from Ancestors genomes. For pairwise comparisons between Cactus and Ancestors-derived reconstructed sequences, we selected full-length sequences generated using the same indel reconstruction method as the corresponding Ancestors progenitors (maximum-likelihood for all except L1MD1).

### ORF refinement and criteria for selection of ORF1 and ORF2 sequences

For each subfamily and genome, we queried all of the ≤100 longest hits >3 kb to identify subsequences homologous to ORF1 and ORF2 using BLASTX against L1HS’s ORF1 and ORF2 protein products: L1RE1 (UniProt: Q9UN81) and LORF2 (UniProt: O00370). For each subfamily, genome, and ORF, we generated a new multiple sequence alignment using MACSE (Ranwez *et al.* 2018). We then submitted these codon-aware ORF1 or ORF2 alignments to FastML v3.11 for ancestral sequence reconstruction using default settings, producing a total of 865 ORF1 reconstructions and 905 ORF2 reconstructions.

For each subfamily, we selected the “best” reconstructed ORF1 and ORF2 on the basis of (1) homology to the expected protein domains and (2) whether those domains occurred in the same reading frame as each other. For (1), we translated the reconstructed ORFs in all 3 forward reading frames, and queried the amino acid sequences against NCBI’s Conserved Domain Database (Lu *et al.* 2020). Homology hits for domains that were truncated (according to CDD’s outputs) or split over multiple reading frames were considered absent (note, however, that CDD allows premature stop codons that are within the domain). For ORF1, we used presence or absence of the C-terminal-most section of the L1 coiled-coil domain (pfam17489), the L1 RRM domain (pfam02994), and the L1 CTD (pfam17490) as selection criteria. For ORF2, we used presence or absence of the Endonuclease (cd09076), RT (cd01650), and DUF1725 [pfam08333—approximately corresponding to a conserved cysteine-rich region (Fanning and Singer 1987)] as selection criteria. For each subfamily and ORF combination (i.e. among all candidates any genome), candidate ORFs were filtered sequentially (1) to first retain only those with the maximum number of domains detected; (2) to retain those in which these domains appear within a minimum number of different reading frames (i.e. included the fewest frame shifts between domains); (3) to retain only those with the smallest number of premature stop codons in the reading frames corresponding to domains; (4) retain those with length nearest to the L1HS-encoded proteins (338aa for ORF1 or 1275aa for ORF2); and (5) to retain those from the oldest genome. The top ranking ORF reconstruction was considered the best for the given subfamily. Note that if only a single candidate emerges at any sorting step, then subsequent steps are not applied.

To generate a single representative “composite sequence” (CS) for each of the 67 L1 subfamilies, we queried the L1HS ORF1 and ORF2 with blastx against the best full-length reconstructed sequences to identify homologous regions. BLAST hits with bit-scores ≥25 and within 400 bp of one another were merged, with the longest contiguous subsection then replaced by the corresponding “best” MACSE-aligned reconstructed ORF from the same subfamily, even if they were derived from different ancestral genomes.

### ORF1 coiled-coil prediction

We scanned translations of the 3 forward frames for each of the best reconstructed ORF1s for predicted coiled-coil formation using EMBOSS pepcoil (Rice *et al.* 2000), which implements the COILS prediction program (Lupas *et al.* 1991). The highest probability coiled-coil prediction in any frame that corresponded to the expected coiled-coil domain region of the ORF was then plotted.

### Analysis of ChIP-seq data for KZFPs

We obtained Position Frequency Matrices (PFMs) for KZFPs from Barazandeh *et al.* (2018) and Lambert *et al.* (2018) (available at humantfs.ccbr.utoronto.ca). We converted the PFMs to position weight matrices (PWMs) using a uniform nucleotide background distribution (the prior nucleotide frequency, used to calculate the log likelihood of each nucleotide at each position of the PWM) of 0.25 and addition of a pseudocount (to avoid zeros in the numerator of log-likelihood ratios) of 0.0001. We then scanned the KZFP PWMs using MOODS (Korhonen *et al.* 2017), with a minimum hit *P*-value threshold of 1E−6 and batch settings. We then compared these results with genomic binding of the corresponding KZFPs to instances of L1 subfamilies in the human genome (Imbeault *et al.* 2017) (from GEO accession GSE78099). L1 subfamilies were considered enriched for in vivo binding by a particular KZFP if (1) they achieved enrichment in genomic loci defined as *P* < 1E−10 (Fisher’s Exact Test, 1-tailed), and (2) at least 10 of the top 500 ChIP-exo peaks for the KZFP overlapped loci corresponding to the L1, identified by RepeatMasker. Since RepeatMasker annotations often fail to assign hits to the subfamily level, instead assigning hits to generalized models, all of the subfamilies corresponding to an enriched generalized model were also considered to be enriched. Correspondences were based upon the L1 hash table found in the RepeatMasker annotation data file, which associates generalized ORF and 5′ UTR models to the subfamily-specific 3′ UTRs. To determine where along the CS models ChIP-seq/exo binding sites occur, peak sequences were extracted for enriched KZFP-subfamily pairs and aligned to the corresponding CS model using mafft –addfragment (Katoh *et al.* 2002). To visualize the relative locations and changes in binding pattern across subfamilies, we remapped KZFP and ChIP-peak coordinates to a Muscle alignment of all 54 CS models.

### Scans of hg38 with CS models

To perform a RepeatMasker scan of hg38, we created a custom library consisting of the 67 CS models and the 20170127 RepeatMasker library, with all L1 class sequences removed. RepeatMasker was run in sensitive mode, with the RMBlast algorithm set as the search engine. We note that the CS models are consensus sequences, not HMMs, and thus the initial RepeatMasker scanning algorithm (RMblast) is different from that used for the Dfam library (nhmmer), and the models themselves lack features such as different weighting on different residues.

To compare RepeatMasker scans to a BLAST search of hg38 with the CS models, we filtered blastn results using an E-value cutoff of 1E−3 for the L1ME subfamilies, and a cutoff of 1E−6 for all newer L1s, as the older sequences are more degenerate and thus tend to possess lower identity to the CS models. The BLAST searches are also complicated by the fact that the L1s are homologous to each other, and are typically fragmented and/or interleaved (problems that RepeatMasker is designed to address). To manage these issues, we adopted a heuristic strategy in which hits for the same L1 subfamily were joined into a single “hit” if within 50 bp of each other. Joined hits from different subfamilies were then grouped if they overlapped by >30 bases (150 bases or greater for joined L1ME hits). For groupings containing hits to more than one L1 model, we chose the hit with the lowest E-value (and highest bit-score, in the event of a tie), and discarded the remainder, even if the overlap is only partial. In a small minority of cases, there were still ties, in which case we made a random selection for construction of figures.

## Results

### L1 RepeatMasker scans across ancestral genomes are consistent with L1 age and species distribution

Our overall approach begins with identification of L1 instances in reconstructed ancestral eutherian genomes. In this study, we used genomes reconstructed with Ancestors 1.1 applied to an alignment of 58 eutherian genomes, extracted from a larger 100 vertebrate genome alignment, and available at http://repo.cs.mcgill.ca/PUB/blanchem/Boreoeutherian/. This software, and the reconstructions, have been previously described (Diallo *et al.* 2010) and evaluated, yielding a 96% accuracy rate on neutrally evolving portions of an ancient eutherian mammal ancestor (Boreoeutherian ancestor) (Blanchette *et al.* 2004). Further improvements in genome alignment, and a larger number of genomes, may improve accuracy and yield of ancient L1 instances, although the recently described Progressive Cactus (Armstrong *et al.* 2020) offered very minimal advantage in our analyses (see below).

**Fig. 2.**
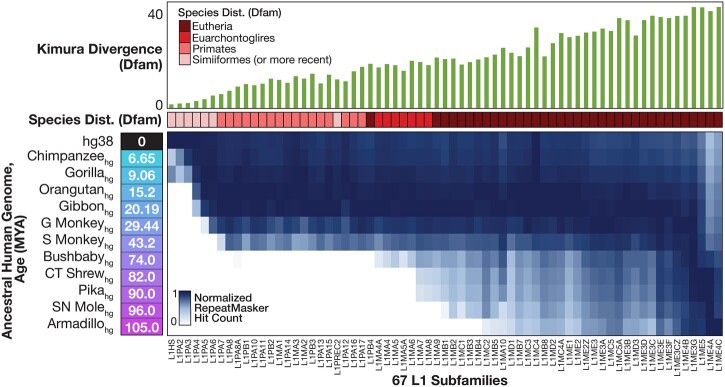
Occurrence of L1 subfamilies in ancestral genomes. Heatmap shows count of each Dfam subfamily in each ancestral genome, normalized to the column maximum. Phylogenetic distance (Kimura divergence) within human and species distribution are taken directly from Dfam.

We used RepeatMasker with Dfam HMM models to obtain L1 elements in each of the 12 genomes (11 reconstructed ancestral genomes + hg38; Fig. 2, heat map at bottom). The distribution of elements across the genomes is almost perfectly consistent with Dfam’s reported average sequence divergence between subfamily member instances in hg38 (at the 3′ end) (Fig. 2, top), as well as the distribution of each subfamily across extant species also as reported by Dfam. For example, primate-distributed subfamilies are exclusively found in genomes younger than the common ancestor of primates (i.e. younger than Bushbaby_hg_, which indicates the common ancestor of human and bushbaby) (Fig. 2, bottom), and these subfamilies typically show lower divergence from their 3′ end consensus model than subfamilies distributed within Euarchontoglires (primates and rodents) or Eutheria (Fig. 2, top).

**Fig. 3.**
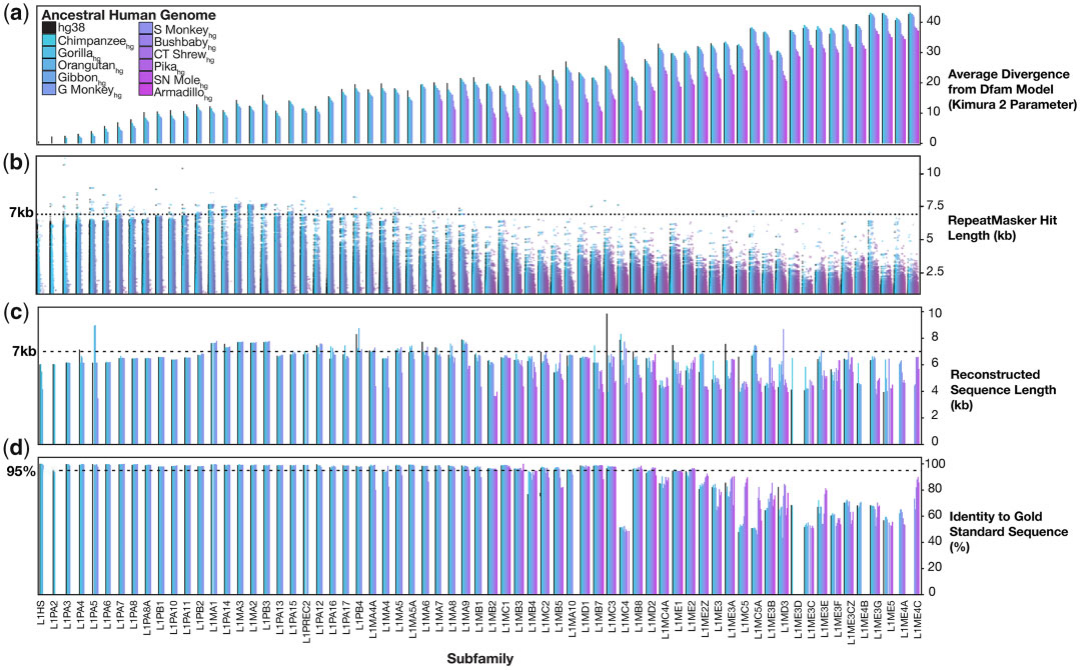
Characteristics of RepeatMasker hits and full-length reconstructed sequences. a) Average phylogenetic distance of detected L1 hits from RepeatMasker consensus models, for each subfamily in each ancestral genome. b) Length of L1 hits for each subfamily in each ancestral genome. c) Length of full-length ancestral reconstructed sequences for every subfamily, in every ancestral genome. d) Percent identity to gold standard sequences for full-length ancestral reconstructed sequences for every subfamily, in every ancestral genome. The highest identity to any gold standard (up to 3 for each subfamily) is shown.

Figure 3 illustrates several global trends in properties of the elements. The L1 instances in older genomes had consistently smaller average Kimura divergence (i.e. adjusted nucleotide substitution rate) from Dfam 3′ end consensus models, compared with those in younger genomes (Fig. 3a), as anticipated (Blanchette *et al.* 2004). The average Kimura divergence does not converge on zero, however, in any cases except the 2 most recent (L1HS and L1PA2). One possible explanation is that the reconstructed genomes contain errors. A second is that none of the reconstructed genomes reflect the exact point at which the elements were active. A third explanation could be that the consensus models are inaccurate, with older subfamilies (right side of Fig. 3) being generally more erroneous. The length distributions of the L1 instances are roughly uniform within subfamilies, regardless of source genome (Fig. 3b). Nonetheless, virtually all L1 subtypes are represented in nearly full-length form (>6 kb) in at least a handful of copies (Fig. 3b).

A caveat of the genome reconstructions we employed is that they are based on a human-referenced alignment, such that elements that are not represented in the human sequence may be omitted from the ancestral genome. While this work was in preparation, an unreferenced alignment algorithm, Progressive Cactus, was described, together with reconstructed ancestral genomes (Armstrong *et al.* 2020). We examined the common eutherian ancestral genome inferred by Cactus, and found that the length distribution of subfamilies in this genome was in fact highly similar to those of hg38 and our human-referenced ancestral genome, although hg38 tended to contain a greater number of elements longer than 3 kb (Supplementary Fig. 2a). In addition, the L1 3′ ends in the Cactus genome reconstruction carry as many mutations as those in our equivalent genome reconstruction (Supplementary Fig. 2b). Below, we also show that L1 elements reconstructed from selected Cactus ancestral genomes are comparable to those obtained from Ancestors 1.1 ancestral genomes. It therefore appears unlikely that repeating the operations described here with the Cactus ancestral genomes would offer dramatic improvement.

### Inference of ancestral L1 elements from Ancestors 1.1 genomes

We next reconstructed a full-length progenitor sequence for each of the 67 L1 subfamilies, from each of the genomes in which it is found (up to 12 genomes). We first recovered the sequences of the 100 longest instances of each subfamily within each genome, and then excluded any that were <3 kb in length. This resulted in a median of 29 input sequences across all genome/subfamily combinations, with a larger number of sequences being available from younger subfamilies and ancestral genomes (all sequences are available on our project website, http://datah.ccbr.utoronto.ca/L1_Reconstruction, or on Zenodo at https://doi.org/10.5281/zenodo.6338536). For each genome and subfamily combination, we then produced a multiple sequence alignment of these ≤100 longest hits using Muscle (Edgar 2004). Each alignment was then supplied to FastML (Ashkenazy *et al.* 2012), which generates a neighbor-joining tree and infers ancestral character states using a maximum-likelihood algorithm and a generalized time reversible (GTR) substitution matrix. Indels were reconstructed using FastML, with either a likelihood-based mixture model or maximum parsimony, thus resulting in 2 sets of reconstructed sequences per alignment. We retained both sets, in anticipation that indels would be prevalent, and that redundancy would therefore be beneficial. This process produced a total of 1,134 reconstructed progenitor sequences (i.e. up to 24 for each of the 67 subfamilies, depending on how many ancestral genomes contain the subfamily).

An overview of the reconstructed sequence lengths and divergence from the “gold standard” consensus is given in Fig. 3, c and d. Here, the “gold standard” is compiled from 3 different data sources [Repbase (Bao *et al.* 2015), Dfam (Hubley *et al.* 2016), and (Khan *et al.* 2006)], such that each subfamily can have up to 3 different gold standard sequences. Among these, only the Khan set are full-length; the gold standard for older L1s is therefore only the 3′ ends (∼900 nt). For younger subfamilies, the reconstructed sequences display approximately similar lengths and identities to gold standards (Khan *et al.* 2006; Hubley *et al.* 2016) across all representative genomes (Fig. 3, c and d, left side), indicating that our methodology broadly reproduces previous outcomes (Khan *et al.* 2006). The reconstructed versions of older L1 subfamilies show greater variability in both length and identity to gold standards. Strikingly, however, those reconstructed from older genomes often show considerable improvement in identity to the gold standard sequence, relative to those built from newer genomes (e.g. L1ME3B, L1ME4C) (Fig. 3, c and d, right side), supporting the validity of the overall approach. We selected a single “best” reconstructed sequence for each L1 subfamily on the basis of its identity to gold standards, and its length. The filtering scheme shown in Supplementary Fig. 3 selects for reconstructed sequences that are among the most similar to the gold standards, and that are closest to (or within) the expected L1 length of 6–8 kb (Supplementary Fig. 4). This process tended to select reconstructed sequences that were derived from older genomes. Overall, ∼87% of all best reconstructed sequences were derived from an ancestral genome rather than hg38, and 79% were within 6–8 kb (Supplementary Fig. 4).

### Comparison to progressive Cactus ancestral genomes and estimate of error rates in reconstructed sequences

The long compute times and manual processing effort associated with our pipeline prohibit us from a global evaluation of alternative ancestral genome reconstruction strategies. Nonetheless, we investigated whether the sequences derived during full-length reconstruction are strongly dependent on the ancestral genome reconstruction method. To this end, we applied our reconstruction pipeline to ancestral genomes from the Progressive Cactus 200-mammal whole-genome alignment (Armstrong *et al.* 2020), with the intention of producing a set of reconstructed sequences complementary to some of the “best” (as defined above) full-length Ancestors-derived progenitors. We selected the subfamilies L1MA2, L1MA4, L1MD1 (derived from the Ancestors 1.1 reconstructed ancestral simian genome), and L1MD2 and 3 (primate), and reconstructed these using the Cactus genomes representing the same ancestor (Supplementary Fig. 5). These subfamilies were selected as test cases because (1) they are ancient (found across all primates, and all eutherian mammals in the cases of L1MD1-3). In addition, (2) the “best” reconstructed sequences derived from the Ancestors genomes are full-length and bear relatively high identity to their reference Repbase 3′ end consensus sequences (∼97–99%), with the exception of L1MD3. L1MD3 serves as a lower-quality reconstructed sequence for comparison.

In general, the Cactus-derived reconstructed sequences demonstrated high sequence identity to the Ancestors-derived L1s over the majority of each element, with the exception of the 5′ ends (Supplementary Fig. 5a). We were intrigued by the low sequence identity over this region, which could be primarily attributable to the ancestral genomes used, or uncertainty introduced by the subsequent L1 reconstruction process. To investigate the latter, we took advantage of the posterior probabilities computed by FastML for all 4 nucleotides at each sequence position (the nucleotide with the highest probability is considered the “maximum-likelihood nucleotide”). Consistent with our expectation and previous findings, the low sequence identity over the 5′ region corresponded to greater uncertainty in the reconstructed L1 sequences, whether those sequences were obtained from Ancestors- or Cactus-derived ancestral genomes (Supplementary Fig. 5a). The uncertainty at a particular position typically coincides with the reduced number of aligned bases in the FastML input alignment (Supplementary Fig. 5b). We note, however, that while the L1s reconstructed from Cactus-derived ancestral genomes generally had a larger number of input sequences (instances greater than the 3 kb inclusion threshold) (Supplementary Fig. 5b), this translated to only marginally higher posterior probabilities over the 5′ ends, with a smaller difference for the older L1MD subfamilies (Supplementary Fig. 5a).

As an additional comparator, we searched the sequences against NCBI’s Conserved Domain Database (CDD) (Lu *et al.* 2020) in all 3 forward frame translations, with the intention of detecting 6 functional domains characteristic of eutherian L1s (the ORF1 coiled-coil domain, RRM and CTD domains, and the ORF2 APE, RT and DUF1725 domains) (Mathias *et al.* 1991; Feng *et al.* 1996; Khazina and Weichenrieder 2009) (Supplementary Fig. 6a). The domains detected for both alternate reconstructed sequences were broadly comparable, with Cactus L1MA4 and L1MD1 containing a single additional domain relative to the Ancestors counterparts, and Cactus L1MD3 containing 2 detectable ORF2 domains. We note that the pipeline’s subsequent ORF reconstruction step addresses this deficit in the full-length reconstructed sequences derived from Ancestors ancestral genomes (see the next section). Thus, while the Progressive Cactus-derived ancestral genomes may offer some improvement, it appears to be marginal, and may be offset by subsequent processing steps.

Given the utility of the FastML posterior probabilities in evaluating reconstructed sequence accuracy, we sought to quantify the uncertainty in the remaining “best” full-length sequences derived from Ancestors genomes. Consistent with expectation, highly divergent older subfamilies were associated with greater uncertainty, i.e. a greater percentage of the sequence is projected to be incorrectly reconstructed (Supplementary Fig. 6b). However, the majority of positions across reconstructed sequences had high posterior probabilities for the maximum-likelihood nucleotide (Supplementary Fig. 6c). The subset of uncertain sites was, as observed in the Cactus comparisons, strongly biased toward the more divergent and frequently truncated 5′ ends for most elements (Supplementary Fig. 6d).

### Codon-aware alignment improves homology to expected protein domains

We next examined the ORFs in the reconstructed L1s. While virtually all of them contained sequences homologous to the Repbase ORF regions, they typically contained frameshifts, which presumably explains the fact that the expected protein domains are not detected (Supplementary Fig. 7, a and b). We reasoned that a codon-aware alignment would reduce the number of frameshifts, and that a simultaneous increase in the expected domain content would be indicative of a bona fide improvement in reconstructing the original sequence. To ask if this is the case, we extracted the ORF1- and ORF2-homologous regions from each of the L1 subfamily instances used in the initial reconstruction step, and aligned them with the codon-aware aligner MACSE (Ranwez *et al.* 2018) prior to conducting ASR with FastML (Supplementary Fig. 8). Briefly, MACSE assumes that all nucleotide sequences in an alignment originated from a common protein-coding ancestor, and extends the classical Needleman−Wunsch alignment generation and scoring method to penalize more severely the introduction of mismatches and indels that introduce amino acid changes or frame shifts. This operation produced a total of 865 reconstructed sequences for ORF1, and 905 for ORF2 (multiple ORF1/2 homologous regions were not detected in all cases). As anticipated, these reconstructed ORFs contained many fewer frameshifts, and were much longer than ORFs detected in the initial reconstructed sequences (Supplementary Fig. 7b). In addition, the expected protein domains were more readily detected, indicating that they are more similar to the ancestral protein sequences (Supplementary Fig. 7b).

The translated ORF protein domain scores and ORF statistics (length and number of frameshifts) provided criteria to select a single representative from among the multiple versions of each reconstructed L1 subfamily (Supplementary Figs. 8 and 9; see *Materials and* *Methods* for details). Briefly, we scanned translations in all 3 reading frames with Conserved Domain Search (Lu *et al.* 2020), and progressively filtered to identify those with the largest number of complete domains, those with the smallest number of ORFs across which complete domains are detected, those with a minimal number of stop codons within the complete ORF, those closest to the ORF lengths of L1HS, and, in the unlikely event of ties, those from older genomes. Reconstructed ORFs were obtained for all subfamilies other than the ancient L1ME3D, for which no ORF2 could be obtained, and L1ME3E, for which neither ORF could be reconstructed.

Of the 66 best reconstructed ORF1s, the vast majority (62/66) contained strong and complete matches to the RRM and CTD domain models (Supplementary Fig. 9). Only 41 were at least 90% of the 338 amino acid expected length for the ORF1p protein, however, with a clear bias toward more recent L1s (Supplementary Fig. 9). In addition, only 33 of the best reconstructed ORF1 proteins exhibit a significant match to the coiled-coil domain (Supplementary Fig. 7c). We suggest that the domain model may not capture all instances of the coiled-coil domain, because virtually all of the reconstructed ORF1 proteins do contain a region that is clearly homologous. This region is computationally predicted to form a coiled-coil in most of the reconstructed ORF1p sequences that were not matches for the coiled-coil domain (Supplementary Fig. 7c).

We also observed that many of the reconstructed ORF1 proteins were truncated at either the N or C terminus (Supplementary Fig. 10). Truncations at the C terminus were short (typically ∼10 AA), and more often found in older L1s (Supplementary Fig. 10b). We believe these represent a technical artifact due to the fact that, in our procedure, ORF1 regions are delineated on the basis of BLAST homology to ORF1 from L1HS (the most recent L1). In support of this notion, DNA corresponding to this region is clearly present in the initial reconstructions of older L1s (e.g. L1MA1, L1MA2) (Supplementary Fig. 11, a and b). Truncations at the N-terminal end of the L1 sequence are typically much longer (Supplementary Figs. 10a and 11c, left) (up to ∼130AA) (note that the L1MA sequences in Khan *et al.* carry these same N-terminal L1 truncations; Supplementary Fig. 11c). We reasoned that truncations at the beginning of ORF1 might be accounted for by the fact that L1 instances in the genome are generally truncated at the 5′ end. Several of the ORF1 proteins missing the N-terminus, however, are from more recent subfamilies (e.g. L1MA2, 3, 4, 5A, 6, 7, 8) which appear to be reconstructed in what is the expected full length, including a 1–2 kb 5′ UTR (Supplementary Fig. 4; also see Supplementary Fig. 12 described below). We note that this region is known to be disordered and poorly conserved [it may have been under diversifying selection multiple times throughout mammalian evolution (Khan *et al.* 2006; Boissinot and Sookdeo 2016)]. It is possible that, as with the C-terminal extension, this region is simply not detected by our initial ORF detection strategy, which relies on homology. Consistent with the poor conservation over the region and the well-characterized variability in mammalian L1 coiled-coil length (Boissinot and Sookdeo 2016), it is also conceivable that these ORF1s contained bona fide truncations (Khazina and Weichenrieder 2009), or underwent a period of intense variability that has reduced alignment quality, as has been documented to have occurred in L1PA7-L1PA3 (Khan *et al.* 2006; Furano *et al.* 2020).

Reconstructed versions of ORF2 more frequently approached the expected length and domain composition: of the 65 that were successfully reconstructed, 60 were at least 90% of the 1,275 amino acid expected length for the ORF2 protein, and 61 exhibited homology to nontruncated copies of both Endonuclease and RT domains (Supplementary Fig. 9).

### Composite ancestral L1s reconstructed from high-scoring subsequences are supported by multiple lines of evidence

To produce a final representative sequence for each L1 subfamily, we identified the regions of the “best” full reconstructed sequences homologous to the L1HS ORF1 and ORF2 using blastx. We then replaced these regions with the same subfamily’s “best” MACSE-aligned reconstructed ORFs. Overall, 65/67 CSs were produced with inserted reconstructed ORF1 and ORF2s (with ORF reconstructions failing for 2 subfamilies from the ancient L1ME3 family, as noted above) (Supplementary Figs. 12 and 13). Most of the CSs contained segments derived from multiple genomes; only 6 were derived entirely from a single genome [L1HS from hg38, L1ME3F from the hominin ancestor (Chimp_hg_), L1MB1 from the hominid ancestor (Orangutan_hg_), and L1PA7, L1PA8, and L1PA11 from the catarrhine ancestor (Green Monkey_hg_)] (Supplementary Fig. 13a).

**Fig. 4.**
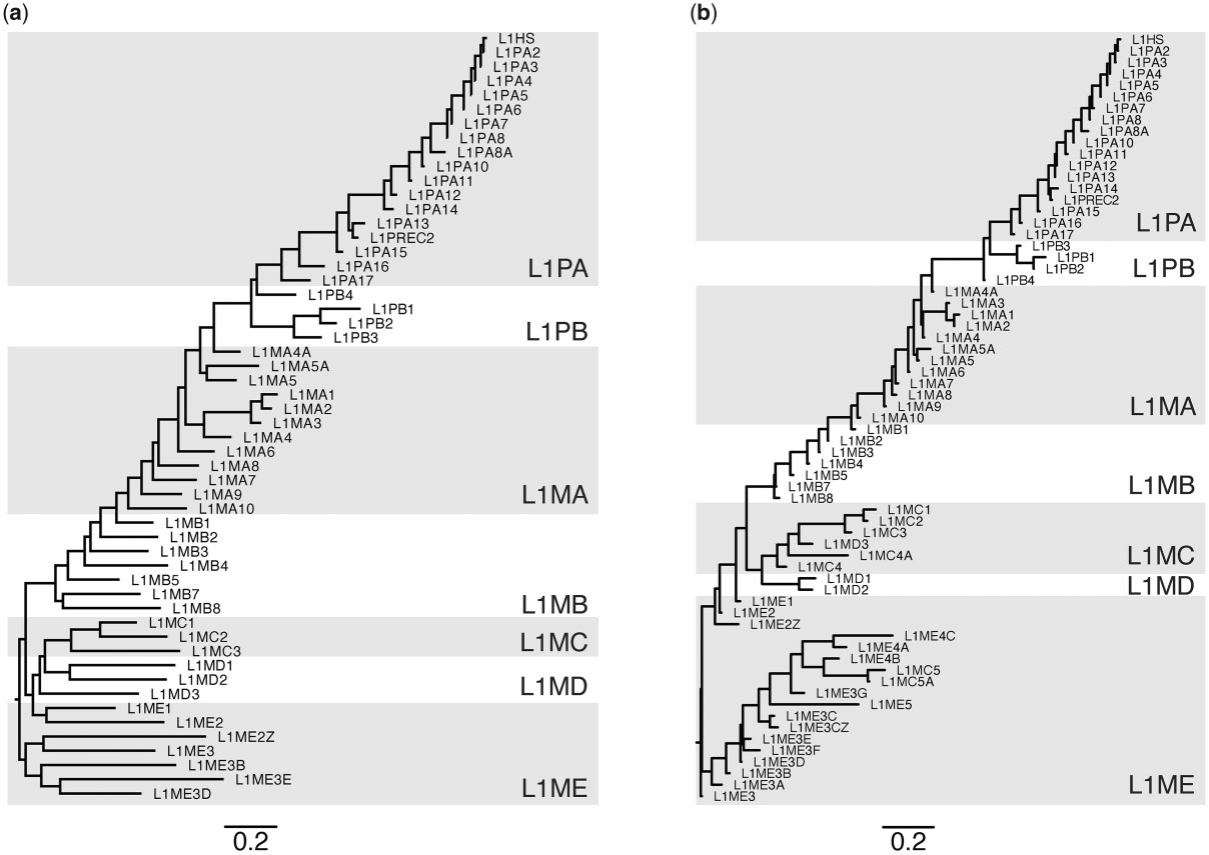
Phylogenetic relationships between CSs, compared with expected subfamily relationships. a) Phylogeny built using a Muscle (Edgar 2004) alignment of full (typically 6–8 kb) CSs for 54 of the 67 L1 subfamilies. The maximum likelihood phylogeny was produced using FastTree (Price *et al.* 2009) with a general time reversible substitution model. b) Phylogeny built using Dfam 3′ end consensus models (median length: 925 bp) for 67 L1 subfamilies, using the same method as (a).

Three independent lines of evidence support the validity of the CSs. First, those representing the most recent L1s (L1HS to L1PA8) contain ORF0, while CSs representing older subfamilies do not, consistent with expectation (Denli *et al.* 2015) (Supplementary Fig. 14). Second, the phylogeny obtained from the full-length CSs largely recapitulates other phylogenetic studies of L1 elements, including the expected ordering from which the nomenclature was derived. An initial phylogeny of the 67 CSs revealed that 13 varied incommensurably from their established phylogenetic relationships (Smit *et al.* 1995) (Supplementary Fig. 15a). These 13 subfamilies all belong to the Eutheria-wide L1MC and L1ME clades, and so represent some of the oldest (and most difficult to confidently reconstruct) subfamilies. These CSs also tended to be derived from many fewer input sequences, were much shorter, and had lower % ID to their respective gold-standards than CSs that did not exhibit drastic differences from the expected phylogeny (Supplementary Fig. 16). When these 13 were removed, the phylogeny of the remaining 54 full-length CSs closely resembles the phylogeny of 67 Dfam 3′ end gold standard consensus models (which are typically composed of ∼700 bp from ORF2, and ∼200 bases of the 3′ UTR) (Fig. 4). Furthermore, the tree topology is consistent with other phylogenetic studies of L1 elements, with L1PA subfamilies evolving as a single lineage, and presumably coexisting with active L1PB elements during early primate evolution (Smit *et al.* 1995; Khan *et al.* 2006).

As recombination of the 5′ UTR is known to have occurred throughout L1 evolution, making the region nonhomologous among various eutherian L1 clades (Khan *et al.* 2006), we produced separate phylogenies based on the different CS components (Supplementary Fig. 15, b and e). A phylogeny of the highly conserved ORF2 largely agreed with those of the full-length CSs and Dfam 3′ ends, while a phylogeny using just ORF1 has a greater number of topological discrepancies, potentially due to the rapid rate of evolution of the ORF1 coiled-coil domain, differing from the more highly conserved C-terminal half of the protein (Boissinot and Furano 2001; Khan *et al.* 2006; Furano *et al.* 2020). In addition, the clades formed in the 5′ UTR phylogeny partially reflect the UTR recruitment scenario posed previously (Khan *et al.* 2006), such as L1PA14-L1HS possessing a similar 5′ UTR, and L1PB3-L1PB1 possessing a unique 5′ UTR (Supplementary Fig. 15c).

**Fig. 5.**
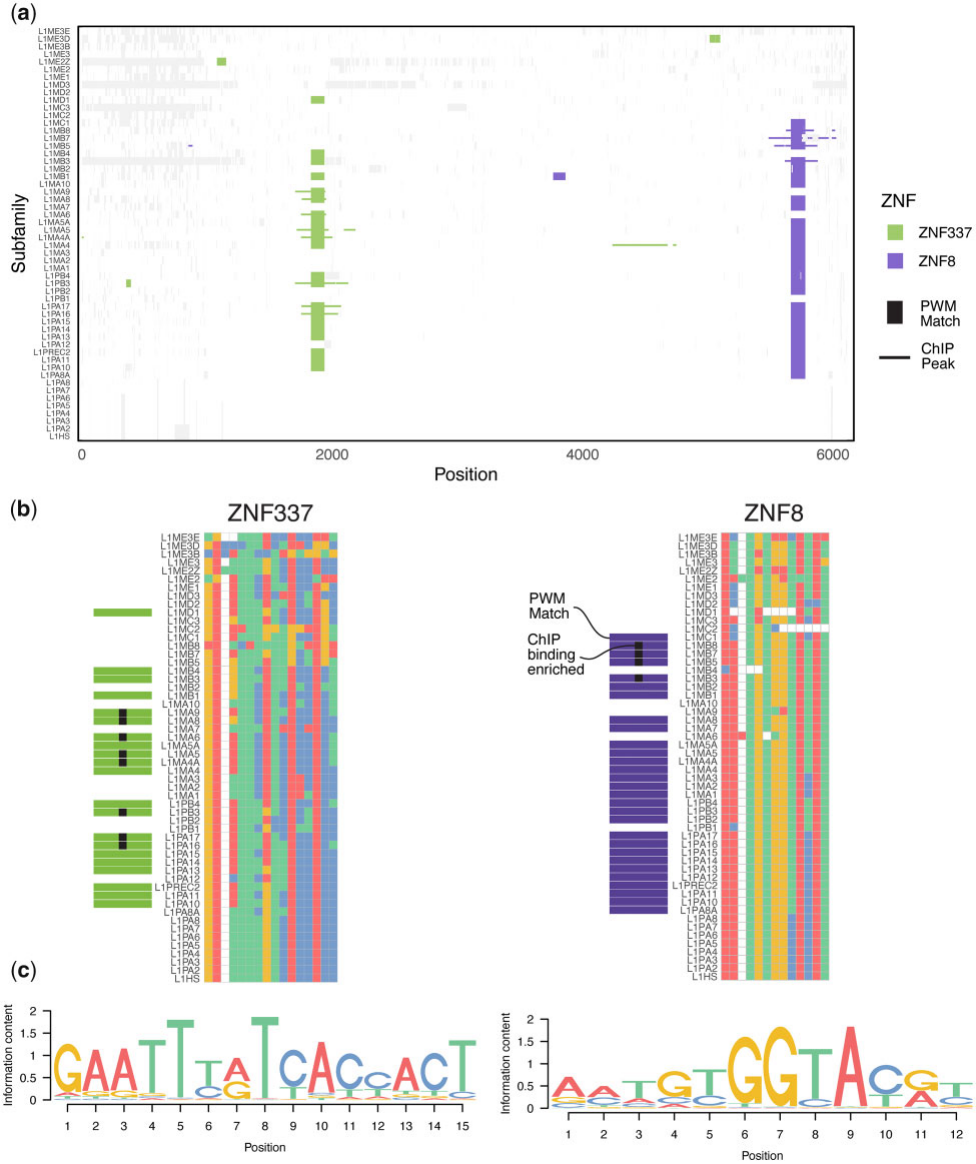
Comparison of KZFP motif matches in reconstructed L1 subfamilies to binding measured by ChIP-seq in human cells. a) Mapping of motif matches (vertical lines) and ChIP-seq/exo peak enrichment (horizontal lines) along an alignment of 54 reconstructed CSs, for the KZFPs ZNF337 and ZNF8. PWM hits are extended by 75 nt on either end, and columns containing large gaps have been trimmed to improve visibility. Aligned bases not overlapping a peak or motif match are represented in white, and gaps in light gray. b) Alignments of ZNF337 (left) and ZNF8 (right) binding sites across subfamilies. Boundaries of alignment correspond to the span of the motifs (shown in c). Subfamilies with motif matches are indicated by horizontal bars to the left of the subfamily labels, and binding enrichment in ChIP data indicated by black squares. A large insertion in L1MA9 has been removed to improve legibility. c) DNA binding motifs of ZNF337 and ZNF8, generated in Barazandeh *et al.* (2018), and derived from ChIP-exo data in Imbeault *et al.* (2017).

A third line of evidence demonstrating that the reconstructed L1 sequences are valid is that they are consistent with gain and loss of PWM motif matches for KZFPs that display differential binding to remnants of the same L1s in the extant human genome. Figure 5 shows 2 examples (ZNF337 and ZNF8) of subfamily-specific gain and loss of motif matches throughout the L1Ms, with corresponding gain and loss of binding enrichment in published ChIP-exo data (Imbeault *et al.* 2017). Unlike the generalized Dfam models, which can represent multiple subfamilies, the reconstructed full-length sequences enable base-level evaluation of gain and loss of binding at individual subfamily resolution. For example, ZNF337 motif matches and ChIP-seq binding enrichment appear to be both lost in L1MA7, but present in both L1MA6 and L1MA8—a subfamily-specific binding site loss that would not be detected based on the single generalized 5′ UTR model in Dfam (Fig. 5b). The changes in nucleotide sequence that underlie loss of binding and/or KZFP hit are generally subtle, single nucleotide changes, unlike the dramatic deletions that underly prior examinations of the arms race model (Jacobs *et al.* 2014).

We note that, at least in the case of ZNF337, the L1 subfamilies significantly bound in ChIP-exo (indicated with black squares) are biased toward C in position 6 of the motif (Fig. 5b), while the motif gives higher weight to a T at this position (Fig. 5c). Motif derivation is empirical, and this particular motif was derived from the same ChIP-exo data analyzed here (Barazandeh *et al.* 2018). Motif searches in which most of the binding sites are retroelements is inherently error-prone, because the sequences are related by common descent, while the motif derivation algorithms assume they are independent. We propose that better consideration of this issue, coupled with knowledge of the progenitor sequences and their phylogeny, could lead to more accurate motif models.

### RepeatMasker searches with reconstructed L1 sequences

**Fig. 6.**
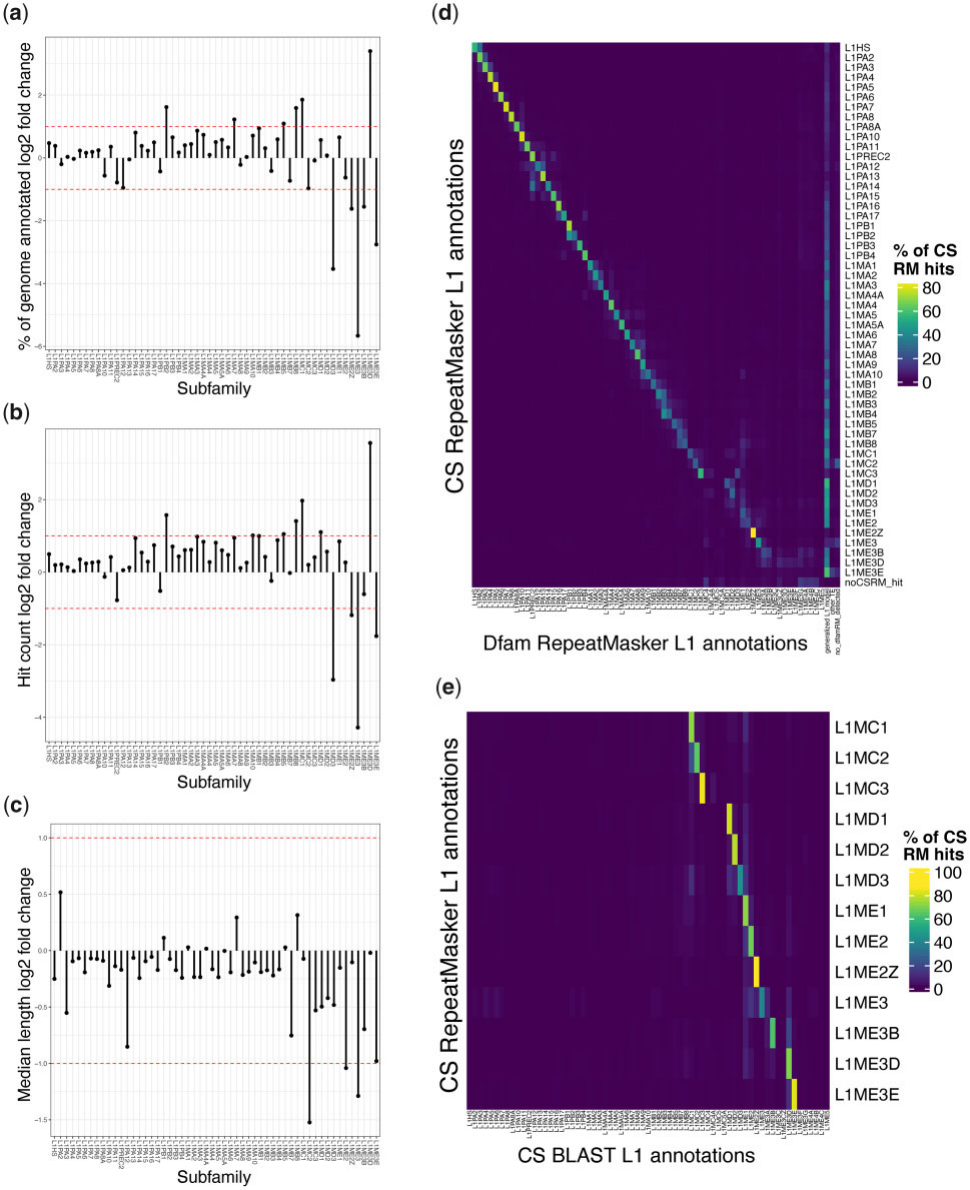
RepeatMasker results of CSs against human genome and comparison to BLAST and RepeatMasker with Dfam. Log2 fold changes, by subfamily, of 3 annotation quality metrics (RepeatMasker with custom CSs library/RepeatMasker with the Dfam 2.0 library). Dotted lines indicate fold changes of 2 and 0.5. The 54 phylogenetically plausible subfamilies are shown. a) Percent coverage of genome. b) Count of individual L1 instances. c) Median lengths of L1 instances. d) Percentage of CS annotations that agreed with an overlapping Dfam annotations. CS annotations that overlapped no Dfam annotations for >5 bp are considered to have “no_dfamRM detected.” e) Percentage agreement between RepeatMasker and CS-called subfamily assignments with BLAST and CS annotations (see also Supplementary Fig. 15).

Finally, as an additional confirmation of the reconstructed L1s, we scanned the human genome (hg38) with all 67 L1 models, using RepeatMasker. Briefly, we created a custom library in which all L1 sequences are removed from the 20170127 RepeatMasker library, and the 67 CS models added (see *Materials and* *Methods* for full details). The 13 CSs lacking phylogenetic support behaved aberrantly in this analysis as well, and results for only the remaining 54 are shown here. Some deviation between the 2 libraries is expected because the CS models are nonprobabilistic single sequences, while the original RepeatMasker Dfam models are HMMs, and thus the primary sequence scan is done differently. Nonetheless, for most of the subfamilies, the proportion of the genome recovered for each, the number of hits, and their lengths were all similar whether we used the default Dfam-based RepeatMasker library (as HMMs), or the custom library with the reconstructed L1 CSs (Fig. 6, a–c). In addition, the specific bases identified for each subfamily typically overlap strongly (Fig. 6d).

The major difference between the CS- and Dfam-based scans was that, almost uniformly, the CS library resulted in elements that were previously ascribed to generalized (i.e. ambiguous) Dfam L1 models being assigned instead to a specific subclass in the CS library (i.e. the overall increase in Fig. 6, a and b, and the vertical line on the right in Fig. 6d). This outcome may be a consequence of the fact that the CS library contains no generalized model(s), but it can also be readily explained by the fact that the subfamily-specific Dfam models are composed exclusively of 3′ ends, and thus have reduced capacity to discriminate between specific subfamilies. The subfamily-specific assignments in CS- vs Dfam-based scans are particularly prominent for older subfamilies (L1MC through L1ME), for which the CS library generally identified fewer hits.

To confirm that this observation is not a consequence of the RepeatMasker scanning methodology, we used BLAST searches to identify sequences in the genome that match the CS models, and parsed the outputs to group overlapping and adjacent matches to assign them to a single best-matching subfamily (Supplementary Fig. 17). The BLAST-based process produced assignments consistent with RepeatMasker outputs with the CS library (Fig. 6e), indicating that the subfamily-specific assignments are a property of the CS sequences, not the search process. Despite the overall reduced sensitivity of the older CS models, we conclude that the reconstructed sequences can empower subfamily-specific assignments for a considerable subset of ancient L1 remnants not currently reflected in mammalian genome annotations. We note that there are a particularly large number of genomic instances corresponding to L1ME3D CS model, which were previously assigned to the generalized RM model (Fig. 6).

## Discussion

We present the first-ever collection of full-length reconstructed L1 sequences spanning human ancestry to the beginning of the eutherian mammals (Supplementary Fig. 18). This collection will be useful in several avenues of further exploration, including more extensive functional analysis of the L1s and their evolution, their interactions with host factors such as C2H2-ZFs, and methodology for repeat annotation of genomes.

We did not undergo a comprehensive exploration of parameter space due to run-time and complexity of the project. As a result, there are a number of potential methodological improvements that could be made to our reconstruction pipeline in future iterations. The ancestral genomes are a foundation of the entire process, but cursory analysis indicated that the new Progressive Cactus reconstruction (Armstrong *et al.* 2020) would not offer dramatic improvement to our process. An obvious alternative source would be the L1 instances found in extant genomes. These sequences would not have the benefit of reduced sequence divergence among input elements conferred by the ancestral genome reconstructions (Fig. 3a), but published resources do contain greater numbers of full-length sequences than any individual reconstructed ancestral genome (Ivancevic *et al.* 2016, 2018), which could potentially compensate.

Technical aspects of the pipeline to explore are also apparent; for example, the reconstructed ancestral coding sequences derived here (and/or those from nonhuman genomes) may be useful for identifying additional L1 instances, and for refinement of ORF ends. The reconstruction process could also leverage existing progenitor models by more heavily weighting sequences that are similar to pre-existing models.

We caution that the reconstructed sequences, particularly those of the older subfamilies, are imperfect. They have not been tested for retrotransposition, and it is unlikely that they would be functional. A recent study found that most tri-alanine mutations in the protein-coding regions of L1HS reduce its function to some degree (Adney *et al.* 2019). The reconstructed 5′ ends of the older reconstructed sequences are inaccurate, which would likely impact the promoter activity. Even if the sequences were completely accurate, retrotransposition assays in human cells would be frustrated by the presence of mechanisms that have evolved to suppress their activity, such the KZFPs. While several resurrected L1PAs successfully retrotranspose in modern or taxonomically distant cells (e.g. mouse) (Ostertag *et al.* 2002; Wagstaff *et al.* 2013; Naufer *et al.* 2016), it remains possible that host factors utilized by the older LINE-1 elements may also be missing from modern mammalian cells, or incompatible due to divergence from their ancestral counterparts. The reduced retrotransposition activity of a resurrected L1PA13A in human and hamster cells relative to a resurrected L1PA8, for example, is potentially due to its maladapted ORF1 protein (Wagstaff *et al.* 2018).

The reconstructed sequences, including the ORFs, largely recapitulate the expected phylogeny, suggesting that they will be useful for examining the evolution of their sequence features. One clear example is the N-terminal coiled-coil domain of ORF1, which does display coiled-coil structural features in the more distant progenitors, despite not matching the domain model. This observation supports the accuracy of the reconstructed sequences, and their utility in investigating evolution (Supplementary Fig. 7c). Recently, the elucidation of a crystal structure for the coiled-coil domain has led to the suggestion that the apparent diversifying selection acting on the coiled-coil domain (Boissinot and Furano 2001; Khan *et al.* 2006) could have instead resulted from compensatory mutations following an initial destabilizing deleterious mutation (Khazina and Weichenrieder 2018). Similarly, analyses of coiled-coil variation in L1PA subfamilies demonstrated that the rapid evolution of this region may be attributable to its genetic robustness, resulting in the persistence of a variety of distinct coiled-coil sequences during primate L1 evolution (Furano *et al.* 2020). The reconstructed sequences described here could be used in experiments to test for self-specificity to evaluate the diversification of the domain, i.e. whether ORF1s prefer to form homo-oligomers. This use-case is also relevant for testing how simultaneously active ORF1ps with distinct coiled-coils avoided hybrid trimer formation, an implication of the mentioned study.

The reconstructed L1s also present an opportunity to isolate the specific sequence changes that led to gain and loss of KZFP binding sites, which are expected to be more subtle overall than the 129 bp deletion eliminating the ZNF93 binding site in L1PA3-L1HS (Jacobs *et al.* 2014; Imbeault *et al.* 2017). Such analyses are complicated by their dependence on accurate models of sequence specificity, which are more difficult to obtain for KZFPs than for other TFs (Barazandeh *et al.* 2018), and may not conform to standard PWM models, if different portions of the C2H2 zinc finger arrays are used at different sites. Still, in the examples of ZNF337 and ZNF8 shown in Fig. 5, a classical motif model almost perfectly explains observed subfamily-specific genomic binding assayed by ChIP-seq.

Finally, the reconstructed L1 sequences may be useful in scanning genomic sequences for transposon remnants. Association of regulatory sites with ancestral retroelements is valuable beyond the academic appeal of mapping evolutionary origins: the elements often contain binding sites for specific regulatory proteins (Kunarso *et al.* 2010), and at some point in their history, the elements had been active in the germline. Our initial finding is that the reconstructed L1s identify many of the same L1-derived elements as the Dfam 2.0 HMM models, and provide a fresh perspective on older L1s. Ultimately, we expect such analyses to further illuminate how this large class of mobile elements—and the largest class of human transcription factors—have evolved to establish the dynamic regulation of the human genome.

## Data availability

All sequences used in reconstructions, the CS models, the data used in constructing figures, and the RepeatMasker results are available on our project website (http://datah.ccbr.utoronto.ca/L1_Reconstruction), or on Zenodo at https://doi.org/10.5281/zenodo.6338536.

Supplemental material is available at *GENETICS* online.

## Supplementary Material

iyac074_Supplementary_Data

## References

[iyac074-B1] Adney EM , Ochmann MT, Sil S, Truong DM, Mita P, Wang X, Kahler DJ, Fenyo D, Holt LJ, Boeke JD. Comprehensive scanning mutagenesis of human retrotransposon LINE-1 identifies motifs essential for function. Genetics. 2019;213(4):1401–1414. 31666291 10.1534/genetics.119.302601 PMC6893370

[iyac074-B2] Alisch RS , Garcia-Perez JL, Muotri AR, Gage FH, Moran JV. Unconventional translation of mammalian LINE-1 retrotransposons. Genes Dev. 2006;20(2):210–224. 16418485 10.1101/gad.1380406 PMC1356112

[iyac074-B3] Armstrong J , Hickey G, Diekhans M, Fiddes IT, Novak AM, Deran A, Fang Q, Xie D, Feng S, Stiller J, et al Progressive Cactus is a multiple-genome aligner for the thousand-genome era. Nature. 2020;587(7833):246–251. 33177663 10.1038/s41586-020-2871-y PMC7673649

[iyac074-B4] Ashkenazy H , Penn O, Doron-Faigenboim A, Cohen O, Cannarozzi G, Zomer O, Pupko T. FastML: a web server for probabilistic reconstruction of ancestral sequences. Nucleic Acids Res. 2012;40(Web Server issue):W580–W584. 22661579 10.1093/nar/gks498 PMC3394241

[iyac074-B5] Bao W , Kojima KK, Kohany O. Repbase update, a database of repetitive elements in eukaryotic genomes. Mob DNA. 2015;6:11. 26045719 10.1186/s13100-015-0041-9 PMC4455052

[iyac074-B6] Barazandeh M , Lambert SA, Albu M, Hughes TR. Comparison of ChIP-Seq data and a reference motif set for Human KRAB C2H2 zinc finger proteins. G3 (Bethesda). 2018;8(1):219–229. 29146583 10.1534/g3.117.300296 PMC5765350

[iyac074-B7] Blanchette M , Diallo AB, Green ED, Miller W, Haussler D. Computational reconstruction of ancestral DNA sequences. Methods Mol Biol. 2008;422:171–184. 18629667 10.1007/978-1-59745-581-7_11

[iyac074-B8] Blanchette M , Green ED, Miller W, Haussler D. Reconstructing large regions of an ancestral mammalian genome in silico. Genome Res. 2004;14(12):2412–2423. 15574820 10.1101/gr.2800104 PMC534665

[iyac074-B9] Boissinot S , Furano AV. Adaptive evolution in LINE-1 retrotransposons. Mol Biol Evol. 2001;18(12):2186–2194. 11719568 10.1093/oxfordjournals.molbev.a003765

[iyac074-B10] Boissinot S , Sookdeo A. The evolution of LINE-1 in vertebrates. Genome Biol Evol. 2016;8(12):3485–3507. 28175298 10.1093/gbe/evw247 PMC5381506

[iyac074-B11] Bruno M , Mahgoub M, Macfarlan TS. The arms race between KRAB-zinc finger proteins and endogenous retroelements and its impact on mammals. Annu Rev Genet. 2019;53:393–416. 31518518 10.1146/annurev-genet-112618-043717

[iyac074-B12] Burton FH , Loeb DD, Voliva CF, Martin SL, Edgell MH, Hutchison CA III. Conservation throughout mammalia and extensive protein-encoding capacity of the highly repeated DNA long interspersed sequence one. J Mol Biol. 1986;187(2):291–304. 3009828 10.1016/0022-2836(86)90235-4

[iyac074-B13] Cao Y , Chen G, Wu G, Zhang X, McDermott J, Chen X, Xu C, Jiang Q, Chen Z, Zeng Y, et al Widespread roles of enhancer-like transposable elements in cell identity and long-range genomic interactions. Genome Res. 2019;29(1):40–52. 30455182 10.1101/gr.235747.118 PMC6314169

[iyac074-B14] Castro-Diaz N , Ecco G, Coluccio A, Kapopoulou A, Yazdanpanah B, Friedli M, Duc J, Jang SM, Turelli P, Trono D. Evolutionally dynamic L1 regulation in embryonic stem cells. Genes Dev. 2014;28(13):1397–1409. 24939876 10.1101/gad.241661.114 PMC4083085

[iyac074-B15] Chuong EB , Elde NC, Feschotte C. Regulatory activities of transposable elements: from conflicts to benefits. Nat Rev Genet. 2017;18(2):71–86. 27867194 10.1038/nrg.2016.139 PMC5498291

[iyac074-B16] Coufal NG , Garcia-Perez JL, Peng GE, Marchetto MC, Muotri AR, Mu Y, Carson CT, Macia A, Moran JV, Gage FH. Ataxia telangiectasia mutated (ATM) modulates long interspersed element-1 (L1) retrotransposition in human neural stem cells. Proc Natl Acad Sci U S A. 2011;108(51):20382–20387. 22159035 10.1073/pnas.1100273108 PMC3251057

[iyac074-B17] de Souza FS , Franchini LF, Rubinstein M. Exaptation of transposable elements into novel cis-regulatory elements: is the evidence always strong? Mol Biol Evol. 2013;30(6):1239–1251. 23486611 10.1093/molbev/mst045 PMC3649676

[iyac074-B18] Denli AM , Narvaiza I, Kerman BE, Pena M, Benner C, Marchetto MC, Diedrich JK, Aslanian A, Ma J, Moresco JJ, et al Primate-specific ORF0 contributes to retrotransposon-mediated diversity. Cell. 2015;163(3):583–593. 26496605 10.1016/j.cell.2015.09.025

[iyac074-B19] Diallo AB , Makarenkov V, Blanchette M. Ancestors 1.0: a web server for ancestral sequence reconstruction. Bioinformatics. 2010;26(1):130–131. 19850756 10.1093/bioinformatics/btp600

[iyac074-B20] Edgar RC. MUSCLE: multiple sequence alignment with high accuracy and high throughput. Nucleic Acids Res. 2004;32(5):1792–1797. 15034147 10.1093/nar/gkh340 PMC390337

[iyac074-B21] Fanning T , Singer M. The LINE-1 DNA sequences in four mammalian orders predict proteins that conserve homologies to retrovirus proteins. Nucleic Acids Res. 1987;15(5):2251–2260. 3562227 10.1093/nar/15.5.2251 PMC340631

[iyac074-B22] Faulkner GJ , Garcia-Perez JL. L1 mosaicism in mammals: extent, effects, and evolution. Trends Genet. 2017;33(11):802–816. 28797643 10.1016/j.tig.2017.07.004

[iyac074-B23] Feng Q , Moran JV, Kazazian HH Jr, Boeke JD. Human L1 retrotransposon encodes a conserved endonuclease required for retrotransposition. Cell. 1996;87(5):905–916. 8945517 10.1016/s0092-8674(00)81997-2

[iyac074-B24] Feschotte C. Transposable elements and the evolution of regulatory networks. Nat Rev Genet. 2008;9(5):397–405. 18368054 10.1038/nrg2337 PMC2596197

[iyac074-B25] Finn RD , Clements J, Eddy SR. HMMER web server: interactive sequence similarity searching. Nucleic Acids Res. 2011;39(Web Server Issue):W29–37. 21593126 10.1093/nar/gkr367 PMC3125773

[iyac074-B26] Furano AV , Jones CE, Periwal V, Callahan KE, Walser JC, Cook PR. Cryptic genetic variation enhances primate L1 retrotransposon survival by enlarging the functional coiled coil sequence space of ORF1p. PLoS Genet. 2020;16(8):e1008991. 32797042 10.1371/journal.pgen.1008991 PMC7449397

[iyac074-B27] Gilbert N , Lutz S, Morrish TA, Moran JV. Multiple fates of L1 retrotransposition intermediates in cultured human cells. Mol Cell Biol. 2005;25(17):7780–7795. 16107723 10.1128/MCB.25.17.7780-7795.2005 PMC1190285

[iyac074-B28] Hohjoh H , Singer MF. Cytoplasmic ribonucleoprotein complexes containing human LINE-1 protein and RNA. EMBO J. 1996;15(3):630–639. 8599946 PMC449981

[iyac074-B29] Hubley R , Finn RD, Clements J, Eddy SR, Jones TA, Bao W, Smit AF, Wheeler TJ. The Dfam database of repetitive DNA families. Nucleic Acids Res. 2016;44(D1):D81–89. 26612867 10.1093/nar/gkv1272 PMC4702899

[iyac074-B30] Imbeault M , Helleboid PY, Trono D. KRAB zinc-finger proteins contribute to the evolution of gene regulatory networks. Nature. 2017;543(7646):550–554. 28273063 10.1038/nature21683

[iyac074-B31] Ivancevic AM , Kortschak RD, Bertozzi T, Adelson DL. Horizontal transfer of BovB and L1 retrotransposons in eukaryotes. Genome Biol. 2018;19(1):85. 29983116 10.1186/s13059-018-1456-7 PMC6036668

[iyac074-B32] Ivancevic AM , Kortschak RD, Bertozzi T, Adelson DL. LINEs between species: evolutionary dynamics of LINE-1 retrotransposons across the eukaryotic tree of life. Genome Biol Evol. 2016;8(11):3301–3322. 27702814 10.1093/gbe/evw243 PMC5203782

[iyac074-B33] Jacobs FM , Greenberg D, Nguyen N, Haeussler M, Ewing AD, Katzman S, Paten B, Salama SR, Haussler D. An evolutionary arms race between KRAB zinc-finger genes ZNF91/93 and SVA/L1 retrotransposons. Nature. 2014;516(7530):242–245. 25274305 10.1038/nature13760 PMC4268317

[iyac074-B34] Jacques PE , Jeyakani J, Bourque G. The majority of primate-specific regulatory sequences are derived from transposable elements. PLoS Genet. 2013;9:e1003504. 23675311 10.1371/journal.pgen.1003504 PMC3649963

[iyac074-B35] Katoh K , Misawa K, Kuma K, Miyata T. MAFFT: a novel method for rapid multiple sequence alignment based on fast Fourier transform. Nucleic Acids Res. 2002;30:3059–3066. 12136088 10.1093/nar/gkf436 PMC135756

[iyac074-B36] Khan H , Smit A, Boissinot S. Molecular evolution and tempo of amplification of human LINE-1 retrotransposons since the origin of primates. Genome Res. 2006;16:78–87. 16344559 10.1101/gr.4001406 PMC1356131

[iyac074-B37] Khazina E , Truffault V, Buttner R, Schmidt S, Coles M, Weichenrieder O. Trimeric structure and flexibility of the L1ORF1 protein in human L1 retrotransposition. Nat Struct Mol Biol. 2011;18:1006–1014. 21822284 10.1038/nsmb.2097

[iyac074-B38] Khazina E , Weichenrieder O. Human LINE-1 retrotransposition requires a metastable coiled coil and a positively charged N-terminus in L1ORF1p. Elife. 2018;7:10.7554/eLife.34960 PMC5940361 29565245

[iyac074-B39] Khazina E , Weichenrieder O. Non-LTR retrotransposons encode noncanonical RRM domains in their first open reading frame. Proc Natl Acad Sci U S A. 2009;106(3):731–736. 19139409 10.1073/pnas.0809964106 PMC2630067

[iyac074-B40] Kolosha VO , Martin SL. In vitro properties of the first ORF protein from mouse LINE-1 support its role in ribonucleoprotein particle formation during retrotransposition. Proc Natl Acad Sci U S A. 1997;94(19):10155–10160. 9294179 10.1073/pnas.94.19.10155 PMC23331

[iyac074-B41] Korhonen JH , Palin K, Taipale J, Ukkonen E. Fast motif matching revisited: high-order PWMs, SNPs and indels. Bioinformatics. 2017;33(4):514–521. 28011774 10.1093/bioinformatics/btw683

[iyac074-B42] Kunarso G , Chia NY, Jeyakani J, Hwang C, Lu X, Chan YS, Ng HH, Bourque G. Transposable elements have rewired the core regulatory network of human embryonic stem cells. Nat Genet. 2010;42(7):631–634. 20526341 10.1038/ng.600

[iyac074-B43] Lambert SA , Jolma A, Campitelli LF, Das PK, Yin Y, Albu M, Chen X, Taipale J, Hughes TR, Weirauch MT. The human transcription factors. Cell. 2018;175(2):598–599. 30290144 10.1016/j.cell.2018.09.045

[iyac074-B44] Lander ES , Linton LM, Birren B, Nusbaum C, Zody MC, Baldwin J, Devon K, Dewar K, Doyle M, FitzHugh W, et al; International Human Genome Sequencing Consortium. Initial sequencing and analysis of the human genome. Nature. 2001;409(6822):860–921. 11237011 10.1038/35057062

[iyac074-B45] Lu S , Wang J, Chitsaz F, Derbyshire MK, Geer RC, Gonzales NR, Gwadz M, Hurwitz DI, Marchler GH, Song JS, et al CDD/SPARCLE: the conserved domain database in 2020. Nucleic Acids Res. 2020;48(D1):D265–D268. 31777944 10.1093/nar/gkz991 PMC6943070

[iyac074-B46] Lupas A , Van Dyke M, Stock J. Predicting coiled coils from protein sequences. Science. 1991;252(5009):1162–1164. 2031185 10.1126/science.252.5009.1162

[iyac074-B47] Martin SL , Cruceanu M, Branciforte D, Wai-Lun Li P, Kwok SC, Hodges RS, Williams MC. LINE-1 retrotransposition requires the nucleic acid chaperone activity of the ORF1 protein. J Mol Biol. 2005;348(3):549–561. 15826653 10.1016/j.jmb.2005.03.003

[iyac074-B48] Mathias SL , Scott AF, Kazazian HH Jr, Boeke JD, Gabriel A. Reverse transcriptase encoded by a human transposable element. Science. 1991;254(5039):1808–1810. 1722352 10.1126/science.1722352

[iyac074-B49] Miao B , Fu S, Lyu C, Gontarz P, Wang T, Zhang B. Tissue-specific usage of transposable element-derived promoters in mouse development. Genome Biol. 2020;21(1):255. 32988383 10.1186/s13059-020-02164-3 PMC7520981

[iyac074-B50] Miller W , Rosenbloom K, Hardison RC, Hou M, Taylor J, Raney B, Burhans R, King DC, Baertsch R, Blankenberg D, et al 28-way vertebrate alignment and conservation track in the UCSC Genome Browser. Genome Res. 2007;17(12):1797–1808. 17984227 10.1101/gr.6761107 PMC2099589

[iyac074-B51] Moran JV , Holmes SE, Naas TP, DeBerardinis RJ, Boeke JD, Kazazian HH Jr. High frequency retrotransposition in cultured mammalian cells. Cell. 1996;87(5):917–927. 8945518 10.1016/s0092-8674(00)81998-4

[iyac074-B52] Naufer MN , Callahan KE, Cook PR, Perez-Gonzalez CE, Williams MC, Furano AV. L1 retrotransposition requires rapid ORF1p oligomerization, a novel coiled coil-dependent property conserved despite extensive remodeling. Nucleic Acids Res. 2016;44(1):281–293. 26673717 10.1093/nar/gkv1342 PMC4705668

[iyac074-B53] Ostertag EM , DeBerardinis RJ, Goodier JL, Zhang Y, Yang N, Gerton GL, Kazazian HH. Jr. A mouse model of human L1 retrotransposition. Nat Genet. 2002;32(4):655–660. 12415270 10.1038/ng1022

[iyac074-B54] Price MN , Dehal PS, Arkin AP. FastTree: computing large minimum evolution trees with profiles instead of a distance matrix. Mol Biol Evol. 2009;26(7):1641–1650. 19377059 10.1093/molbev/msp077 PMC2693737

[iyac074-B55] Ranwez V , Douzery EJP, Cambon C, Chantret N, Delsuc F. MACSE v2: toolkit for the alignment of coding sequences accounting for frameshifts and stop codons. Mol Biol Evol. 2018;35(10):2582–2584. 30165589 10.1093/molbev/msy159 PMC6188553

[iyac074-B56] Rice P , Longden I, Bleasby A. EMBOSS: the European Molecular Biology Open Software Suite. Trends Genet. 2000;16(6):276–277. 10827456 10.1016/s0168-9525(00)02024-2

[iyac074-B57] Scott AF , Schmeckpeper BJ, Abdelrazik M, Comey CT, O'Hara B, Rossiter JP, Cooley T, Heath P, Smith KD, Margolet L. Origin of the human L1 elements: proposed progenitor genes deduced from a consensus DNA sequence. Genomics. 1987;1(2):113–125. 3692483 10.1016/0888-7543(87)90003-6 PMC7135745

[iyac074-B58] Smit AF , Toth G, Riggs AD, Jurka J. Ancestral, mammalian-wide subfamilies of LINE-1 repetitive sequences. J Mol Biol. 1995;246(3):401–417. 7877164 10.1006/jmbi.1994.0095

[iyac074-B59] Suzuki J , Yamaguchi K, Kajikawa M, Ichiyanagi K, Adachi N, Koyama H, Takeda S, Okada N. Genetic evidence that the non-homologous end-joining repair pathway is involved in LINE retrotransposition. PLoS Genet. 2009;5(4):e1000461. 19390601 10.1371/journal.pgen.1000461 PMC2666801

[iyac074-B60] Swergold GD. Identification, characterization, and cell specificity of a human LINE-1 promoter. Mol Cell Biol. 1990;10(12):6718–6729. 1701022 10.1128/mcb.10.12.6718-6729.1990 PMC362950

[iyac074-B61] Tempel S. Using and understanding RepeatMasker. Methods Mol Biol. 2012;859:29–51. 22367864 10.1007/978-1-61779-603-6_2

[iyac074-B62] Villar D , Berthelot C, Aldridge S, Rayner TF, Lukk M, Pignatelli M, Park TJ, Deaville R, Erichsen JT, Jasinska AJ, et al Enhancer evolution across 20 mammalian species. Cell. 2015;160(3):554–566. 25635462 10.1016/j.cell.2015.01.006 PMC4313353

[iyac074-B63] Wagstaff BJ , Kroutter EN, Derbes RS, Belancio VP, Roy-Engel AM. Molecular reconstruction of extinct LINE-1 elements and their interaction with nonautonomous elements. Mol Biol Evol. 2013;30(1):88–99. 22918960 10.1093/molbev/mss202 PMC3525338

[iyac074-B64] Wagstaff BJ , Wang L, Lai S, Derbes RS, Roy-Engel AM. Reviving a 60 million year old LINE-1 element. Gene Rep. 2018;11:74–78. 30221208 10.1016/j.genrep.2018.02.007 PMC6136441

