# Supplementary material for: Reconstruction of full-length LINE-1 progenitors from ancestral genomes
Source: Genetics. 2022 May 12;221(3):iyac074. doi: 10.1093/genetics/iyac074 (PMC9252281; doi:10.1093/genetics/iyac074)
Supplement: iyac074_Supplementary_Data [file iyac074_supplementary_data.pdf]

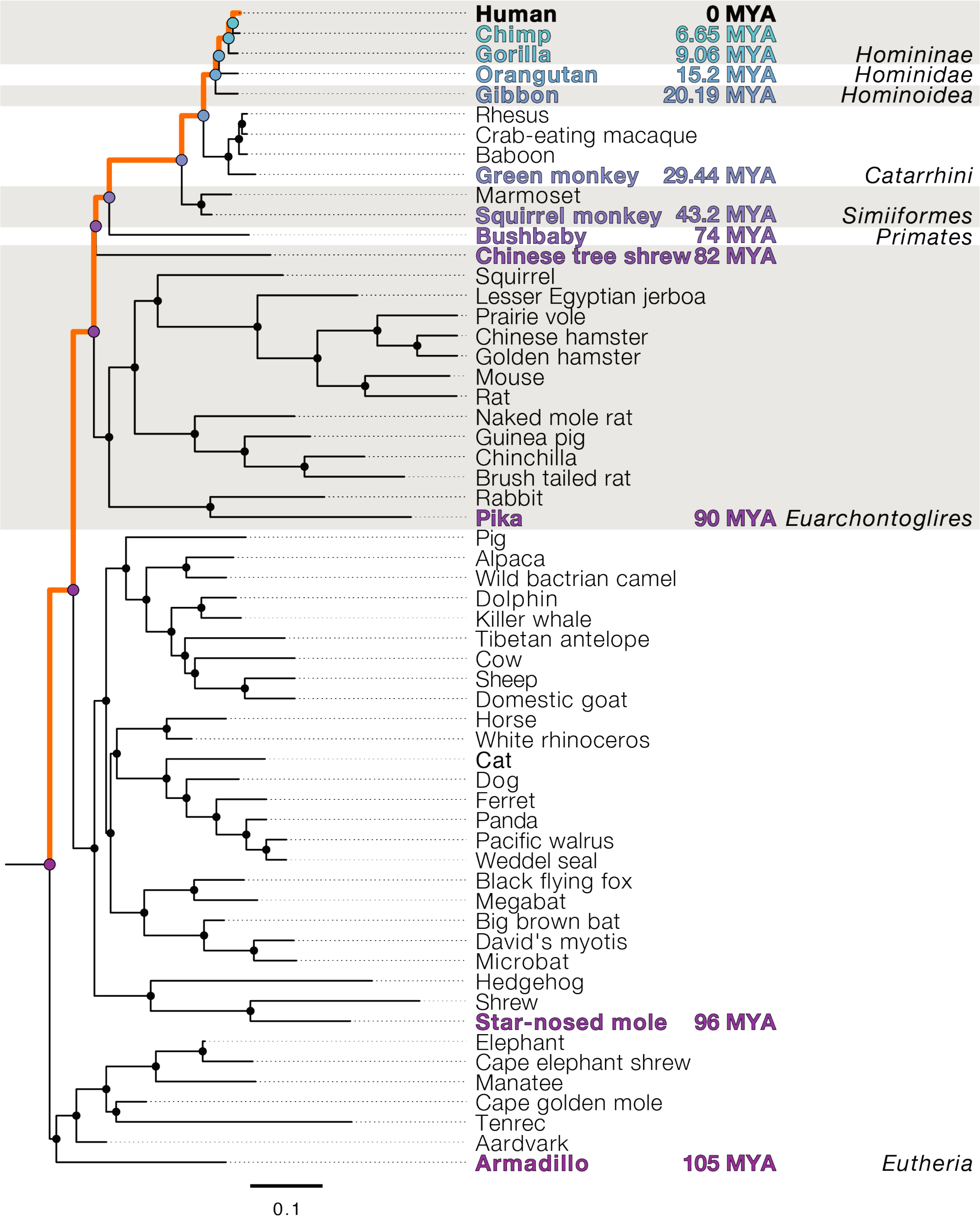

Figure S1. Overview of reconstructed ancestral human genomes.
Phylogeny of extant genomes used to produce ancestral reconstructed genome sequences. Coloured internal nodes represent the ancestral human genomes in the dataset. Coloured text corresponding to node colours indicates the extant species with which the ancestral genome is a common ancestor to human, and estimated divergence time from human in millions of years ago (MYA), taken from TimeTree (Kumar et al. 2017). The orange line indicates the human lineage that is reconstructed.

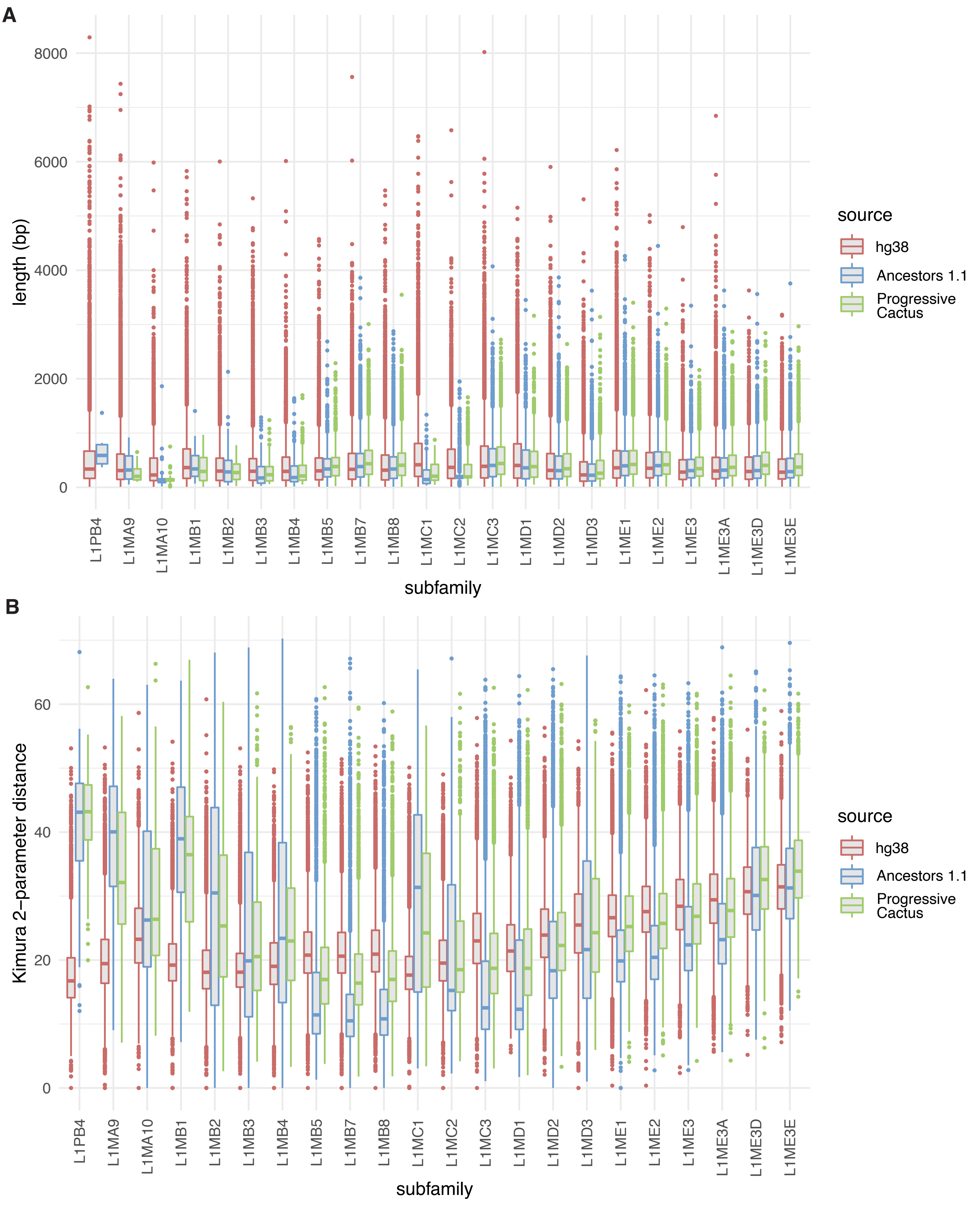

Figure S2. Comparison of RepeatMasker outputs for L1 subfamilies from the modern human genome (hg38) and two alternative reconstructed pan-Eutherian genomes (Ancestors 1.1 (this study) and Progressive Cactus (Armstrong et al. 2020).

All genomes were annotated using the RepeatMasker and the Dfam 2.0 library. L1 subfamilies shown are those distributed across Eutheria, according to Dfam annotations.

A. Distributions of lengths among detected elements.

B. Distributions of Kimura 2-parameter distances among detected elements.

Create 1134 ancestral reconstructed sequences

(12 genomes x 3 ≤ 67 subfamilies x 2 FastML methods)

Get percent identity between each reconstructred sequence and subfamily gold standards

MUSCLE align reconstructed sequences to gold standard consensus sequences from Dfam, Repbase, and Khan *et al* 2006

Consider only reconstructed sequences with high identity to gold standards

For each subfamily, discard reconstructed sequence-gold standard pairs where identity is not within 1.5% of the highest reconstructed sequence-gold standard identity in the same subfamily

	ID	Length
RS1	0.988	6242
RS2	0.995	6299
RS3	0.962	6725
RS4	0.922	6101
RS5	0.991	8225
RS6	0.996	5143

Normalize lengths

Consider equivalent equal all lengths 6-8kb and discard reconstructed sequences with lengths >8kb

	ID	Length
RS1	0.988	6000
RS2	0.995	6000
RS5	0.991	8225
RS6	0.996	5143

Select longest sequence with highest identity to a gold standard

Rank order reconstructed sequences by normalized length, then by gold standard identity, and select the top sequence

	ID	Length
RS2	0.995	6000
RS1	0.988	6000
RS6	0.996	5143

Figure S3. Process for selecting 'Full-length' reconstructed sequences.

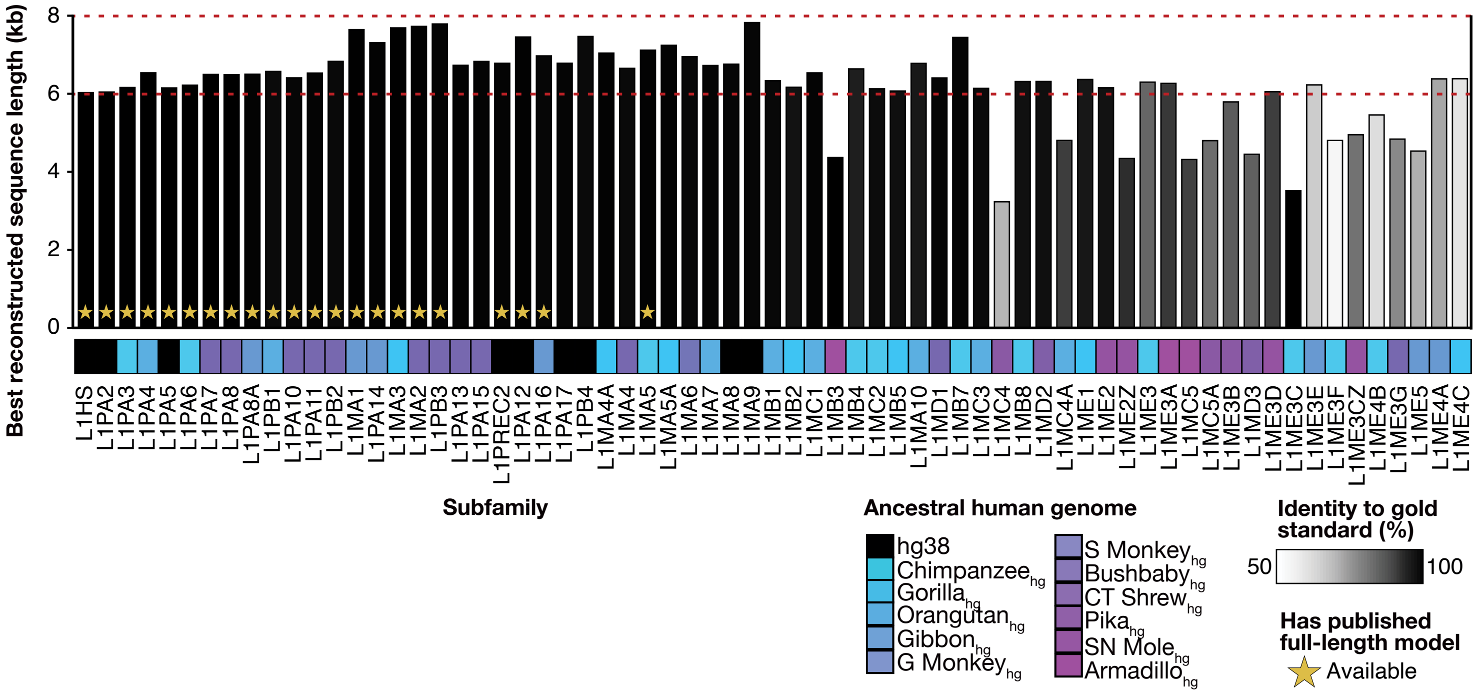

Figure S4. Lengths and source genomes of best full-length reconstructed sequence for each subfamily.
Methods for conducting the reconstructions and selecting the best reconstructed sequence for each subfamily are illustrated in **Figure S3** and described in **Methods**.

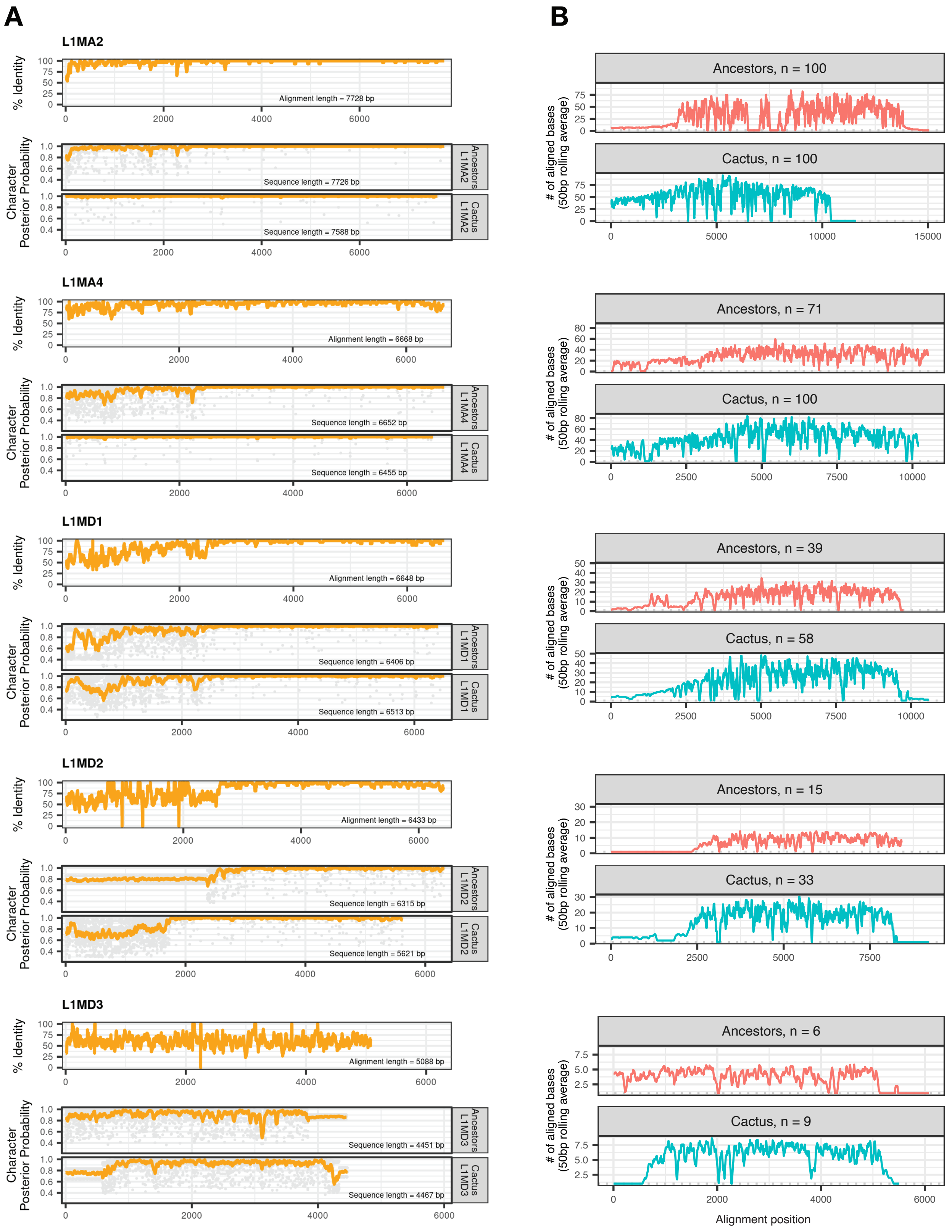

Figure S5. Comparison of full-length L1 reconstructed sequences derived from ancestral whole genomes reconstructed with Ancestors 1.1 or Progressive Cactus.

A. % identity panels: 30 nt rolling average of the percent identity between the Ancestors genome-derived “best” full-length reconstructed sequences, and the reconstructed sequence derived from the corresponding Cactus ancestral genome (simian for L1MA2, L1MA4, and L1MD1, and primate for L1MD2-3), and using the same indel-reconstruction method. Percent identity was calculated on the pairwise alignment, excluding internal gap positions. Posterior probability panel sets: Maximum-likelihood posterior probabilities across sequence positions for the two alternate reconstructed sequences (grey dots). The 30 nt rolling average is given as an orange line.

B. 50 bp rolling average of the number of base pairs aligned at each position in the Muscle source alignments that served as input to FastML. The number of input sequences in the alignment are given in the grey strips.

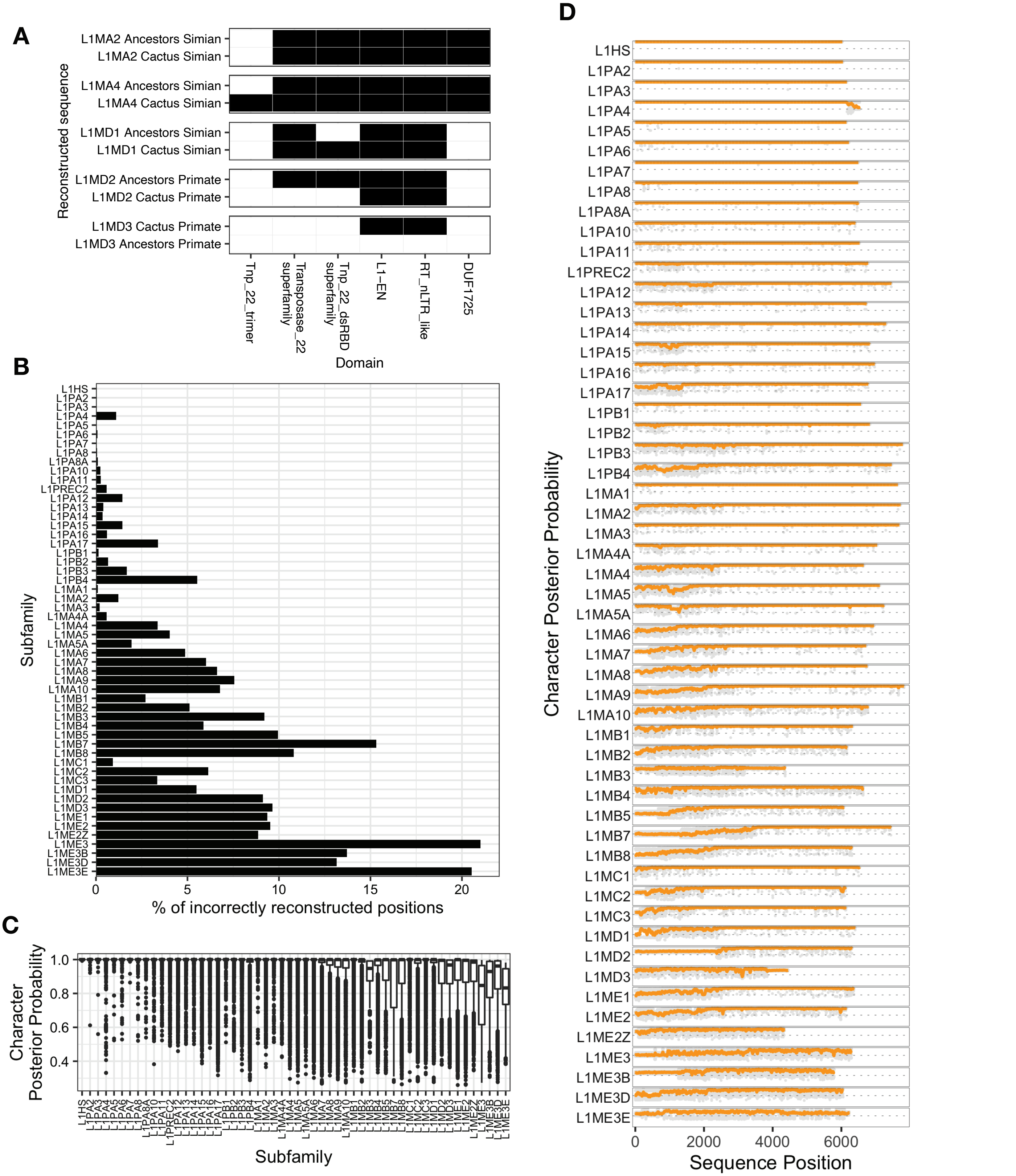

Figure S6. Conserved Domain searches of Cactus-derived reconstructed sequences, and FastML reconstruction uncertainty of "best" full-length sequences.

A. Conserved Domain search of the compared Ancestors or Cactus ancestral genome-derived reconstructed L1 sequences. Presence (black)/absence (white) of L1 protein-coding domains are shown, as detected by CD search on of three-frame translations of the full-length sequences.

B. Percentage of reconstructed L1 sequence positions that are the incorrect nucleotide. Percentages were calculated by dividing the cumulative posterior-probabilities of non-maximum likelihood nucleotides within a full-length reconstructed sequence by the total sequence length.

C. Distribution of all maximum-likelihood nucleotide posterior probabilities.

D. Maximum-likelihood posterior probabilities across sequence positions for each "best" full-length sequence (grey dots). the top of each bar represents a probability of 1.0, and the bottom a probability of 0; the dotted line is a reference for 0.6. The rolling average using a window of 30 nt is given as an orange line.

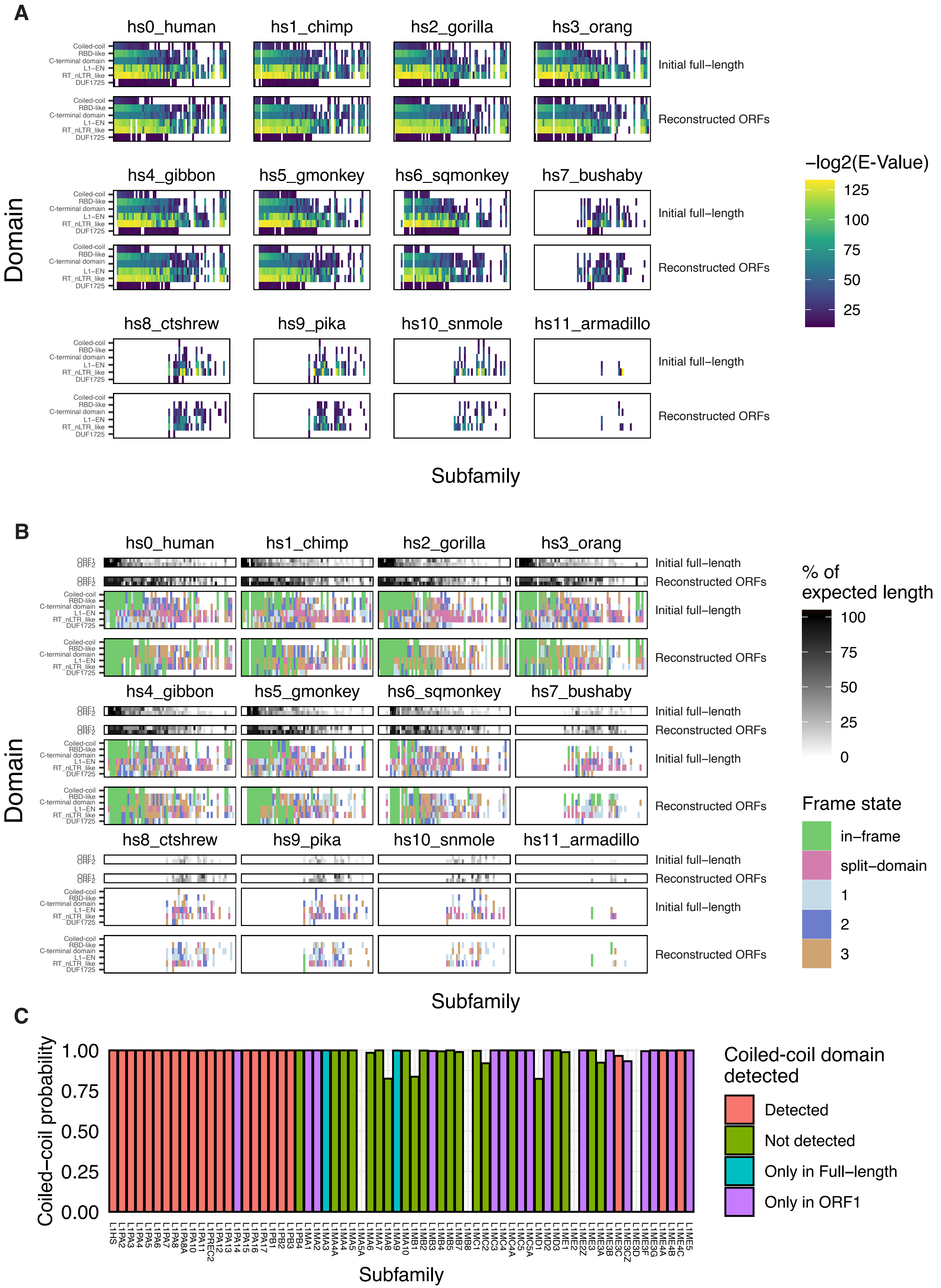

A
hs0_human
hs1_chimp
hs2_gorilla
hs3_orang
hs4_gibbon
hs5_gmonkey
hs6_sqmonkey
hs7_bushaby
hs8_ctshrew
hs9_pika
hs10_snmole
hs11_armadillo
Coiled-coil
RBD-like
C-terminal domain
L1−EN
RT_nLTR_like
DUF1725
Initial full−length
Reconstructed ORFs
Domain
Subfamily
−log2(E-Value)
125
100
75
50
25
B
ORF1
ORF2
% of expected length
100
75
50
25
0
Frame state
in−frame
split−domain
1
2
3
C
Coiled−coil probability
1.00
0.75
0.50
0.25
0.00
L1HS
L1PA2
L1PA3
L1PA4
L1PA5
L1PA6
L1PA7
L1PA8
L1PA8A
L1PA10
L1PA11
L1PREC2
L1PA12
L1PA13
L1PA14
L1PA15
L1PA16
L1PA17
L1PB1
L1PB2
L1PB3
L1PB4
L1MA1
L1MA2
L1MA3
L1MA4
L1MA4A
L1MA5
L1MA5A
L1MA6
L1MA7
L1MA8
L1MA9
L1MA10
L1MB1
L1MB2
L1MB3
L1MB4
L1MB5
L1MB7
L1MB8
L1MC1
L1MC2
L1MC3
L1MC4
L1MC4A
L1MC5
L1MC5A
L1MD1
L1MD2
L1MD3
L1ME1
L1ME2
L1ME2Z
L1ME3
L1ME3A
L1ME3B
L1ME3C
L1ME3CZ
L1ME3D
L1ME3F
L1ME3G
L1ME4A
L1ME4B
L1ME4C
L1ME5
Subfamily
Coiled-coil domain detected
Detected
Not detected
Only in Full−length
Only in ORF1

Figure S7. Comparison of results from NCBI Conserved Domain Search of reconstructed sequences.

A. Heatmaps showing CDS -log2 E-values of the three ORF1 and three ORF2 domains. Each pair of heatmaps represents a genome, with the top heatmap representing the full-length reconstructed sequences derived from that genome, and the lower heatmap the reconstructed ORFs. Subfamilies (ordered youngest to oldest) run along the x-axis of each heatmap. White cells indicate that no domain was detected.

B. Black and white bars: Lengths of the longest ORFs (as detected by NCBI ORFfinder) that correspond to the ORF1 (top row) and ORF2 (bottom row) coding sequences, as a % of the expected lengths (based on the UniProt L1HS ORF1 and ORF2). The upper bar contains the longest ORFs of the initial full-length reconstructed sequences, and the lower bar are those found in the reconstructed ORFs. Subfamilies are ordered along the x-axis as in Panel A, and are aligned with those in the coloured heatmaps.

Coloured heatmaps: Cell colours represent the frame distributions of detected domains, with green indicating different domains belonging to the same ORF as being in frame with one another, pink representing a domain split across multiple frames, and the remaining colours (1, 2, 3) representing domains that were detected out of frame from the others detected in that ORF.

C. Bar height represents the probability of coiled-coil formation in the best reconstructed ORF1 sequence for a given subfamily. Bar fill colour represents the presence of the coiled-coil domain in both the best full-length and reconstructed ORF1s (red), just the reconstructed full-length sequence (blue), just the reconstructed ORF1 (purple), or neither (green).

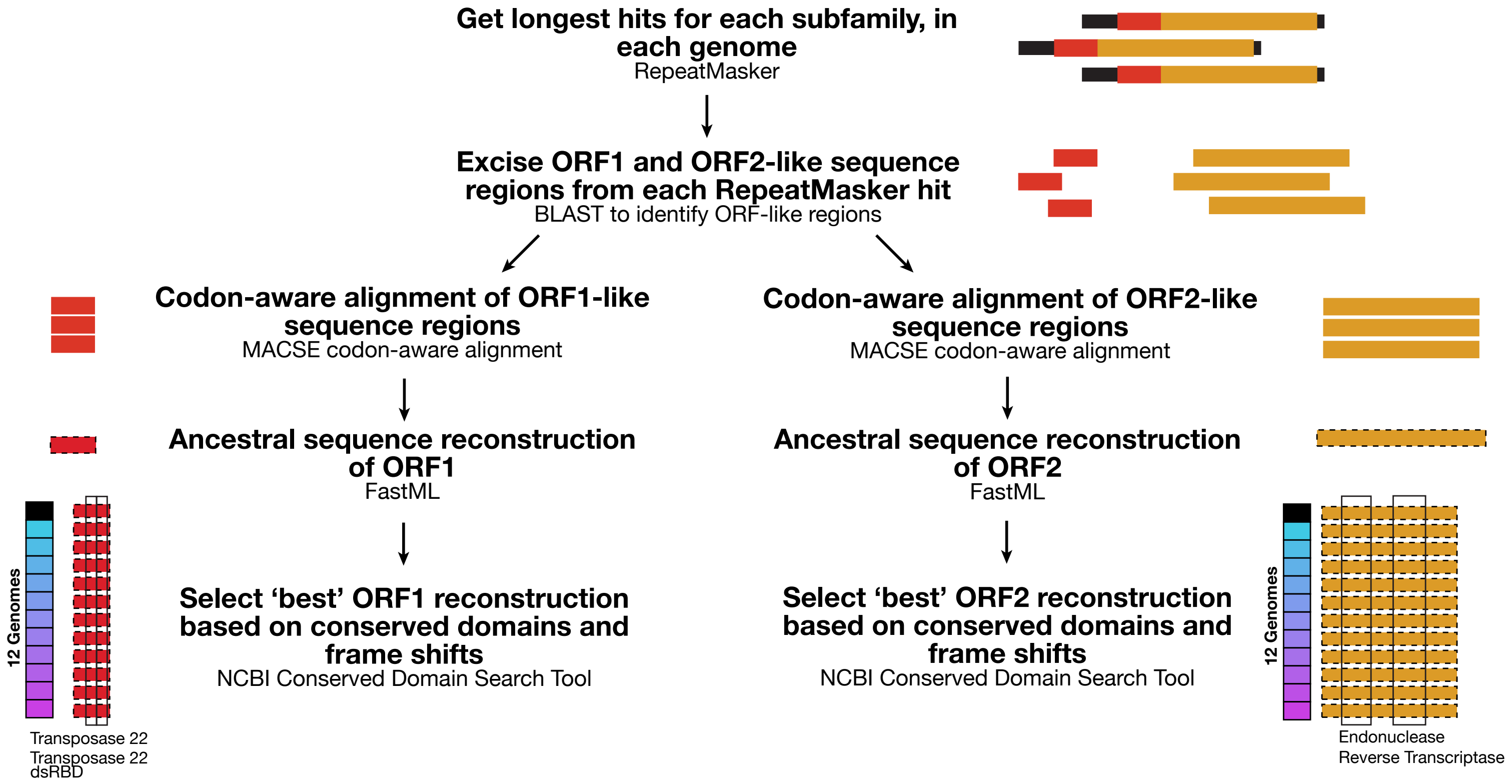

Figure S8. Schematic of the ORF reconstruction method and selection of best reconstructed ORFs.
See **Methods** for detailed steps.

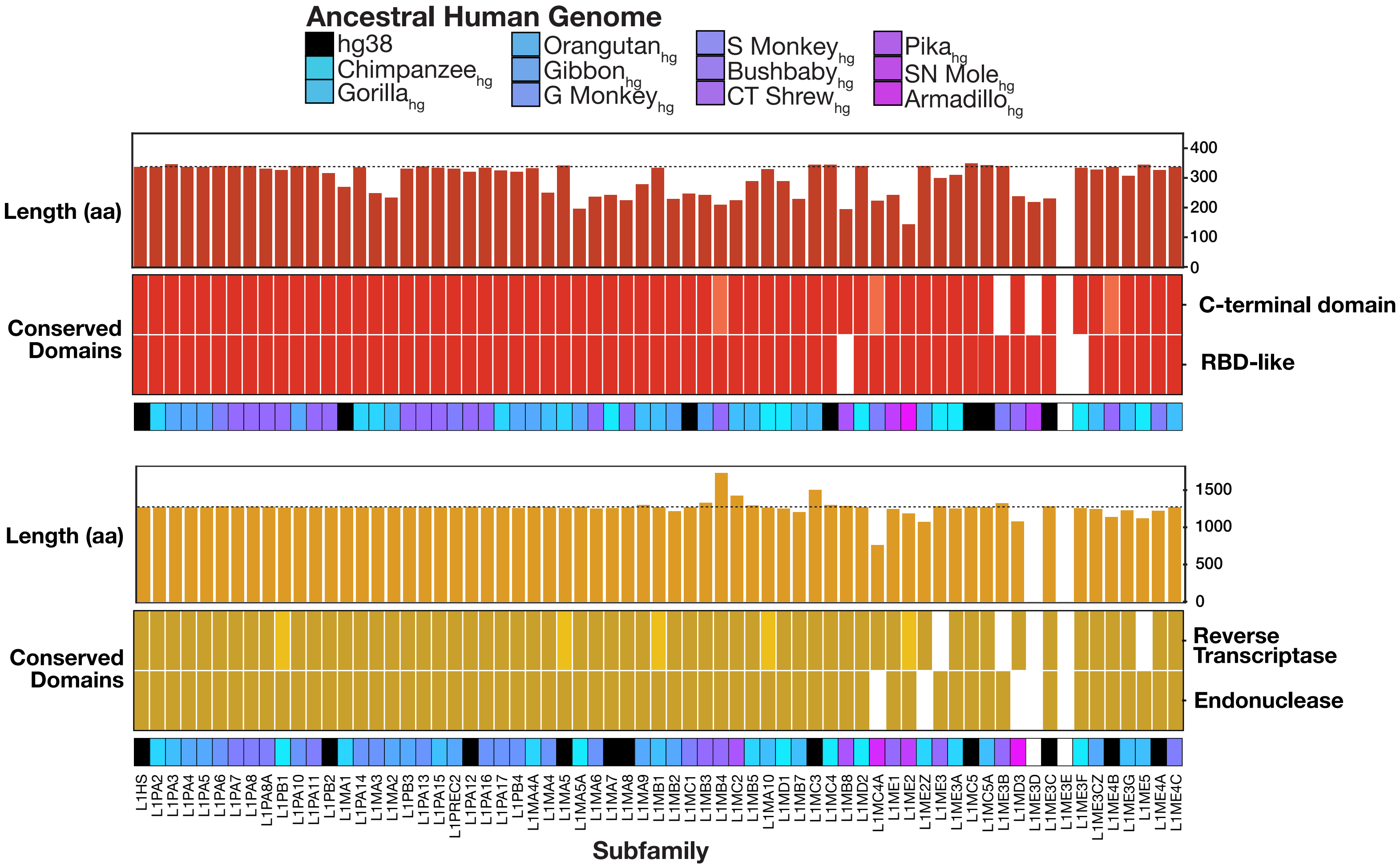

Figure S9. Properties of the best reconstructed ORF1 and ORF2 sequences for each L1 subfamily.

Colour changes on the 'Conserved Domains' heatmap indicate a frame shift between the two domains of the same protein. Dotted lines on each bar graph represent the expected length of each ORF, based on the L1HS proteins as deposited in UniProt (ORF1: Q9UN81, ORF2: O00370).

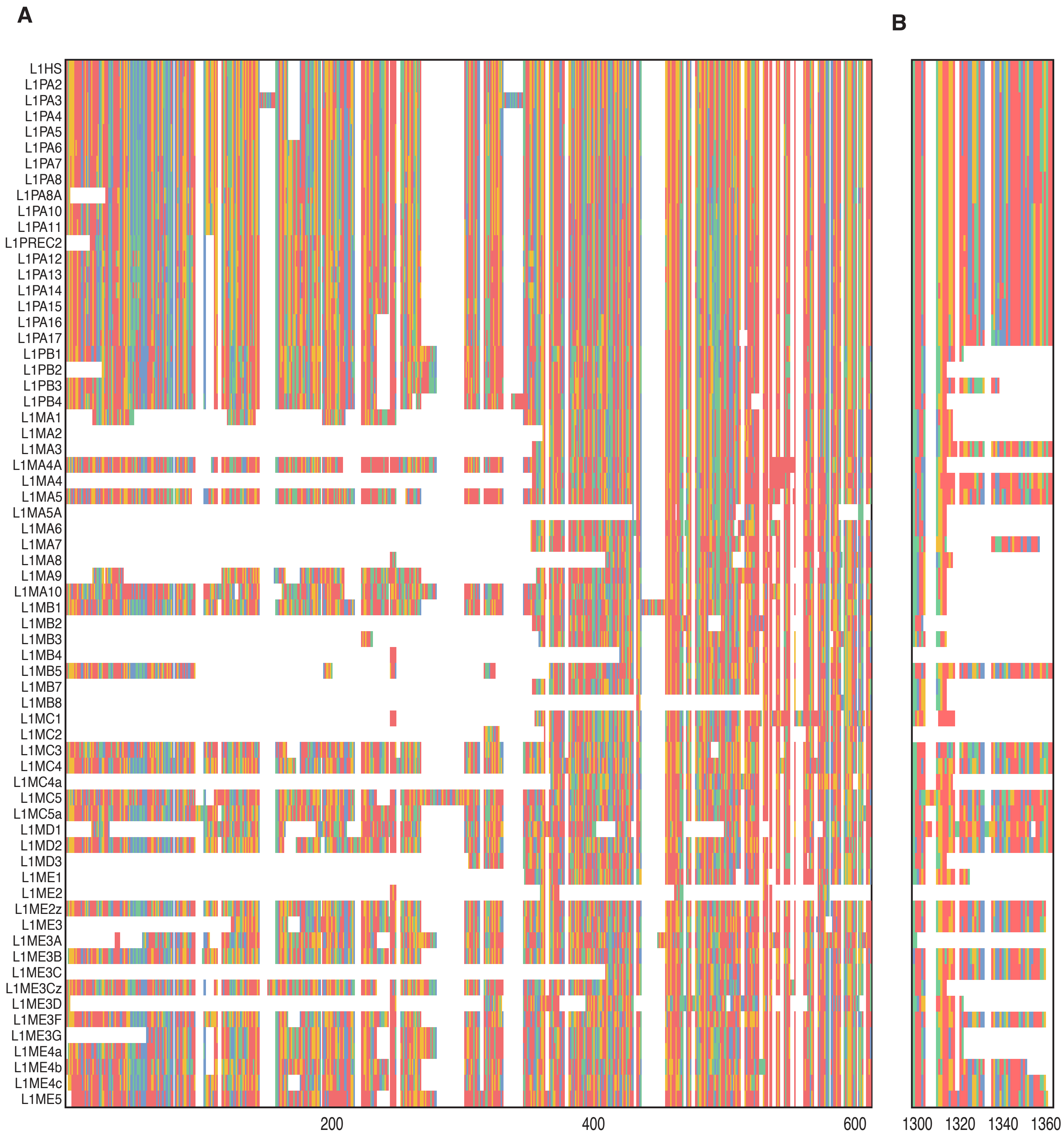

Figure S10. N-terminal and C-terminal truncations in the reconstructed ORF1.
Muscle multiple sequence alignment is shown for the best reconstructed ORF1s, highlighting:
A. The N-terminal/5’ end, through the coiled-coil domain.
B. The C-terminal/3’ extension region.

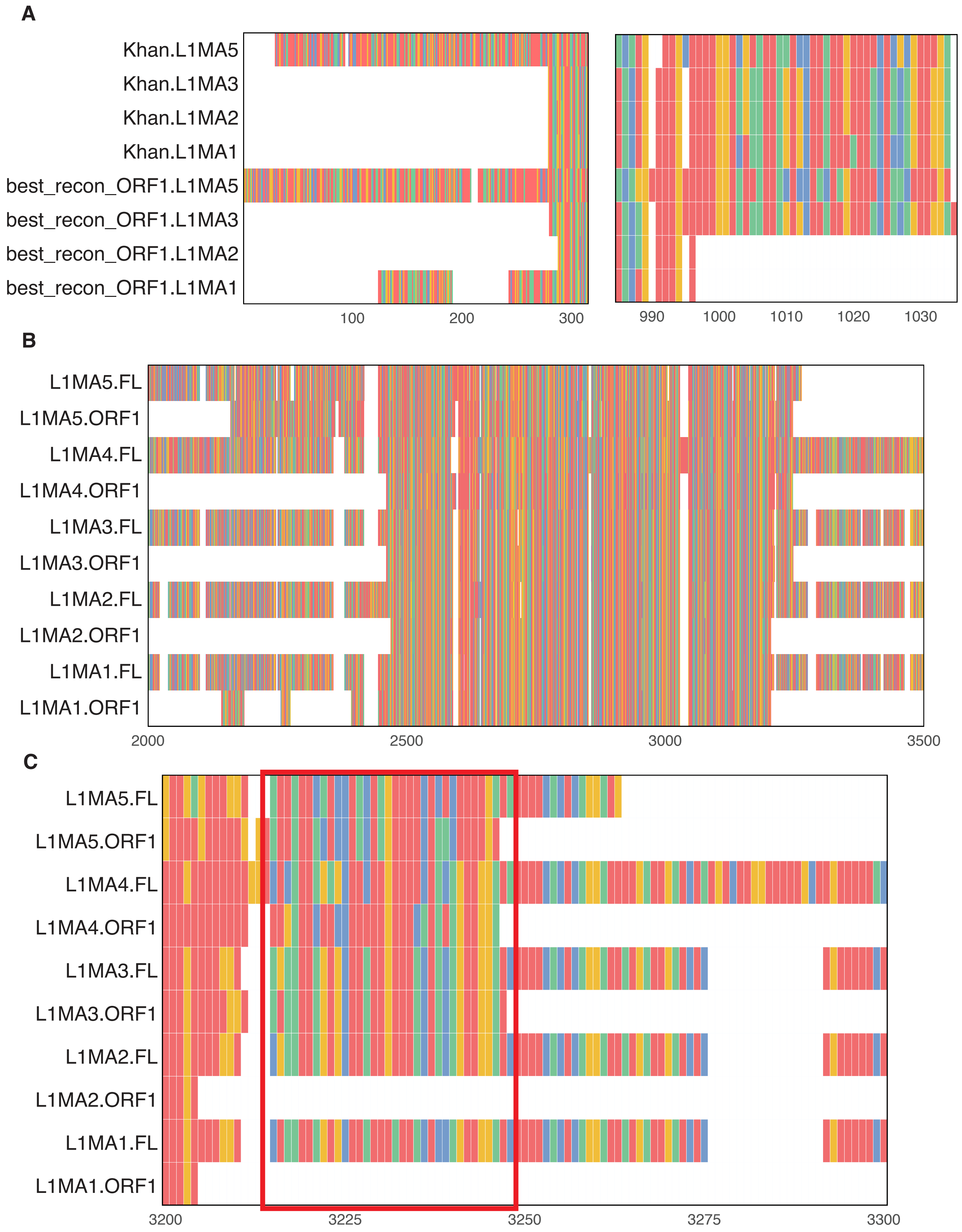

Figure S11. C-terminal extensions of reconstructed L1MA ORF1s.
A. Alignment of reconstructed L1MA5-1 ORF1s, with the corresponding best reconstructed full-length sequences.
B. Left: N-terminal alignment of ORF1 sequences from Khan et al. 2006, and the best reconstructed ORF1 sequences. Right: C-terminus alignments of these sequences.
C. Alignment from B, restricted to the C-terminal extension, highlighted with a red box.

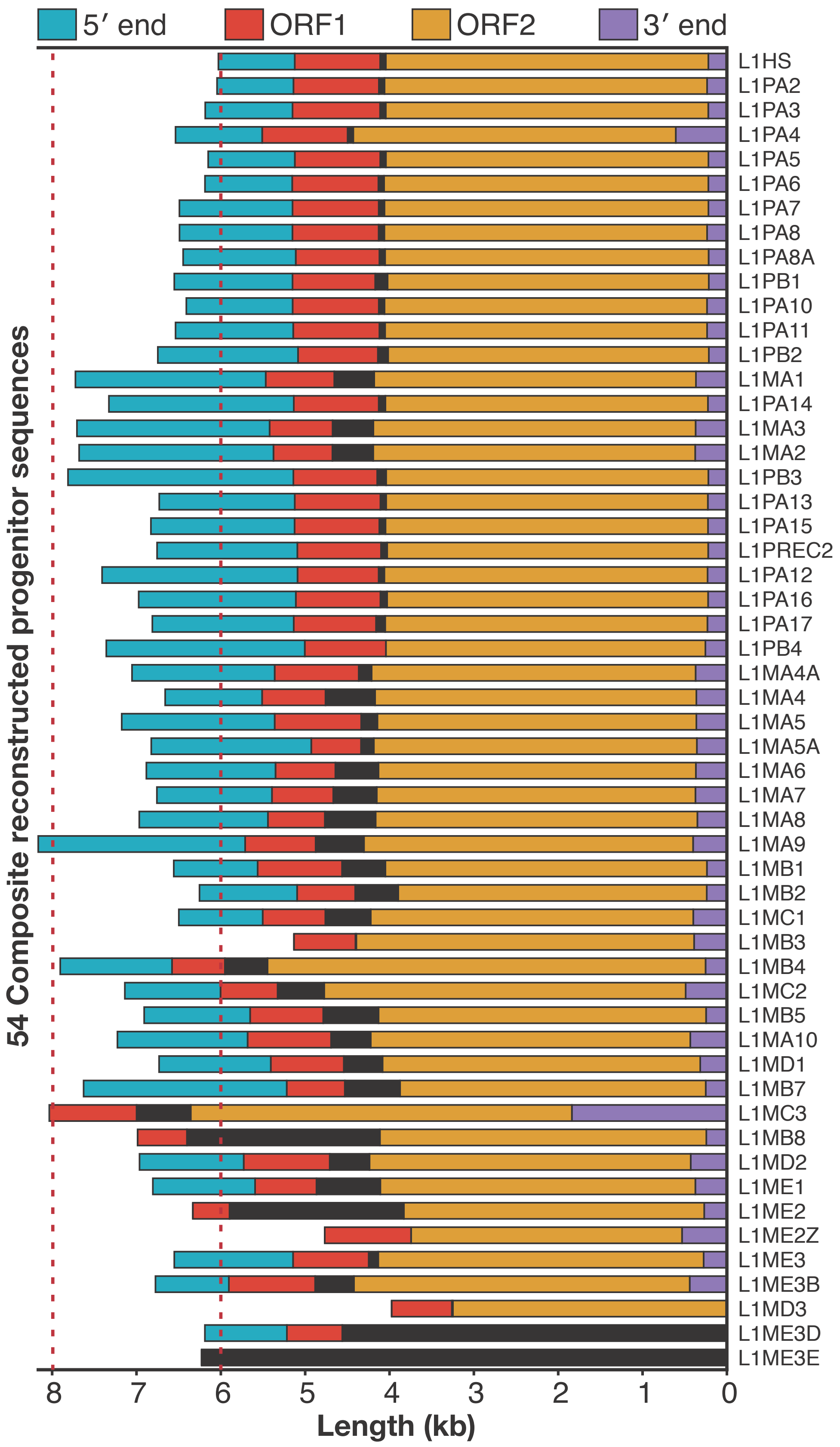

Figure S12. Lengths of Composite Sequence components.
Includes only the 54 subfamilies that produced successful Composite Sequences.

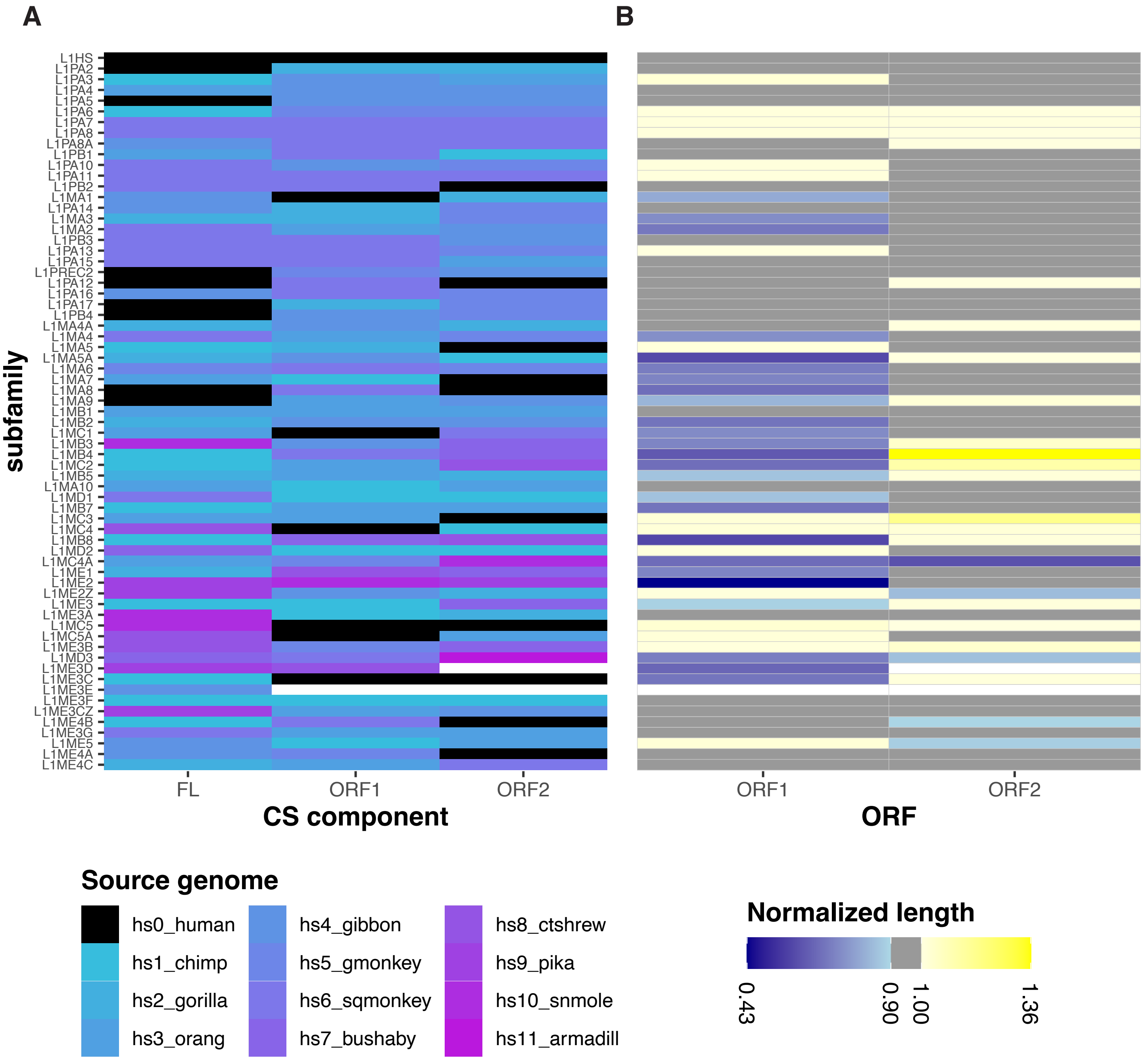

Figure S13. Composite Sequence component sources and lengths.
A. Source genomes of the full-length (FL) reconstructed sequences, and the two reconstructed ORFs that comprise each of the best Composite Sequences. Each genome is named according to the most distant species relative to humans that share the common ancestral genome. ORFs for which no successful reconstructed sequences were produced are shown in white.
B. Lengths of the best reconstructed ORF1s and ORF2s, normalized to a proportion of the corresponding L1HS proteins (aa lengths). ORFs between 90-100% of the length of the L1HS proteins are grey.

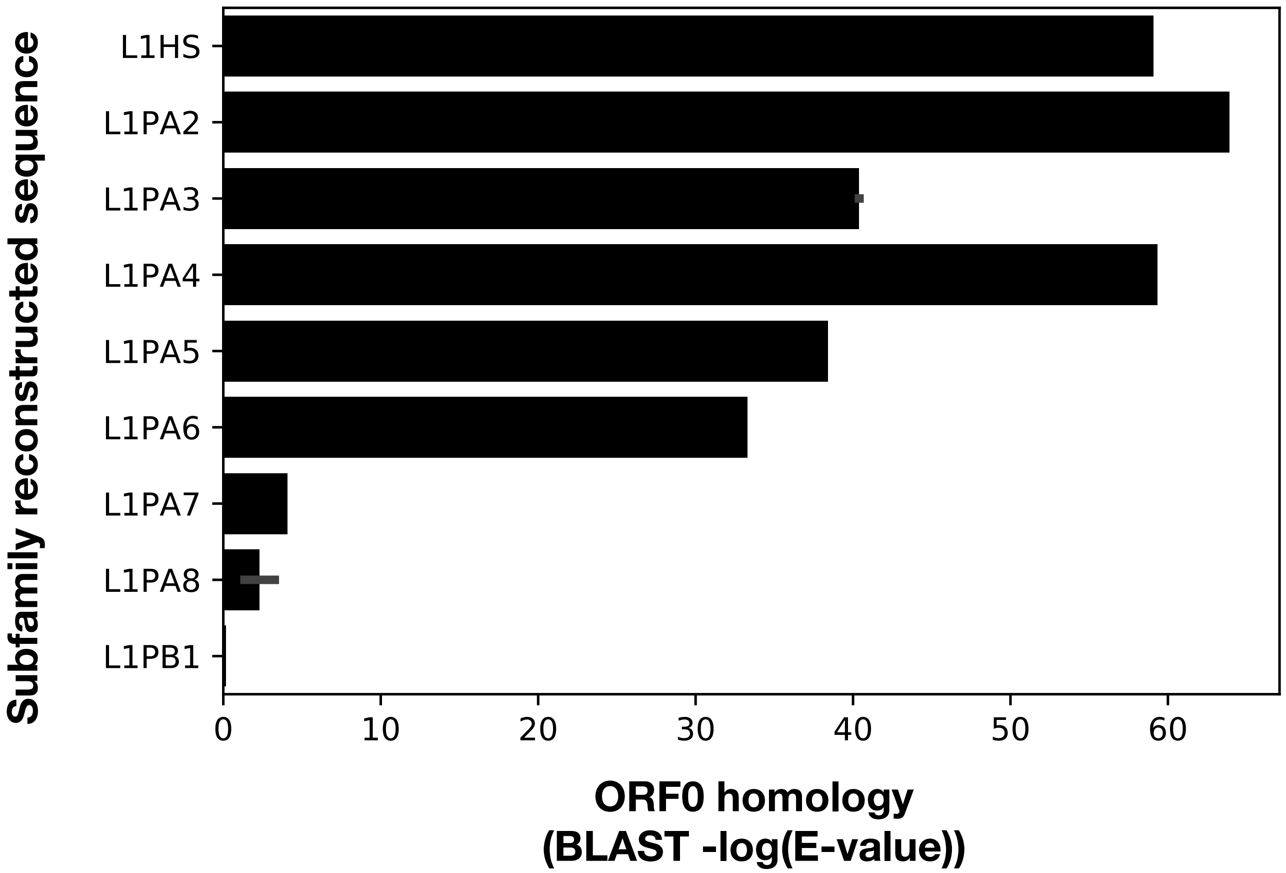

Figure S14. ORF0 homology detected in full-length reconstructed progenitor sequences.

TBLASTN scores for the ORF0 amino acid sequence from (Denli et al. 2015), searched against the Composite Sequences. Denli *et al.* (2015) reported that ORF0 was detected in L1PA8-L1HS. Subfamily progenitor sequences with BLAST bit-scores < 25 for ORF0 are excluded.

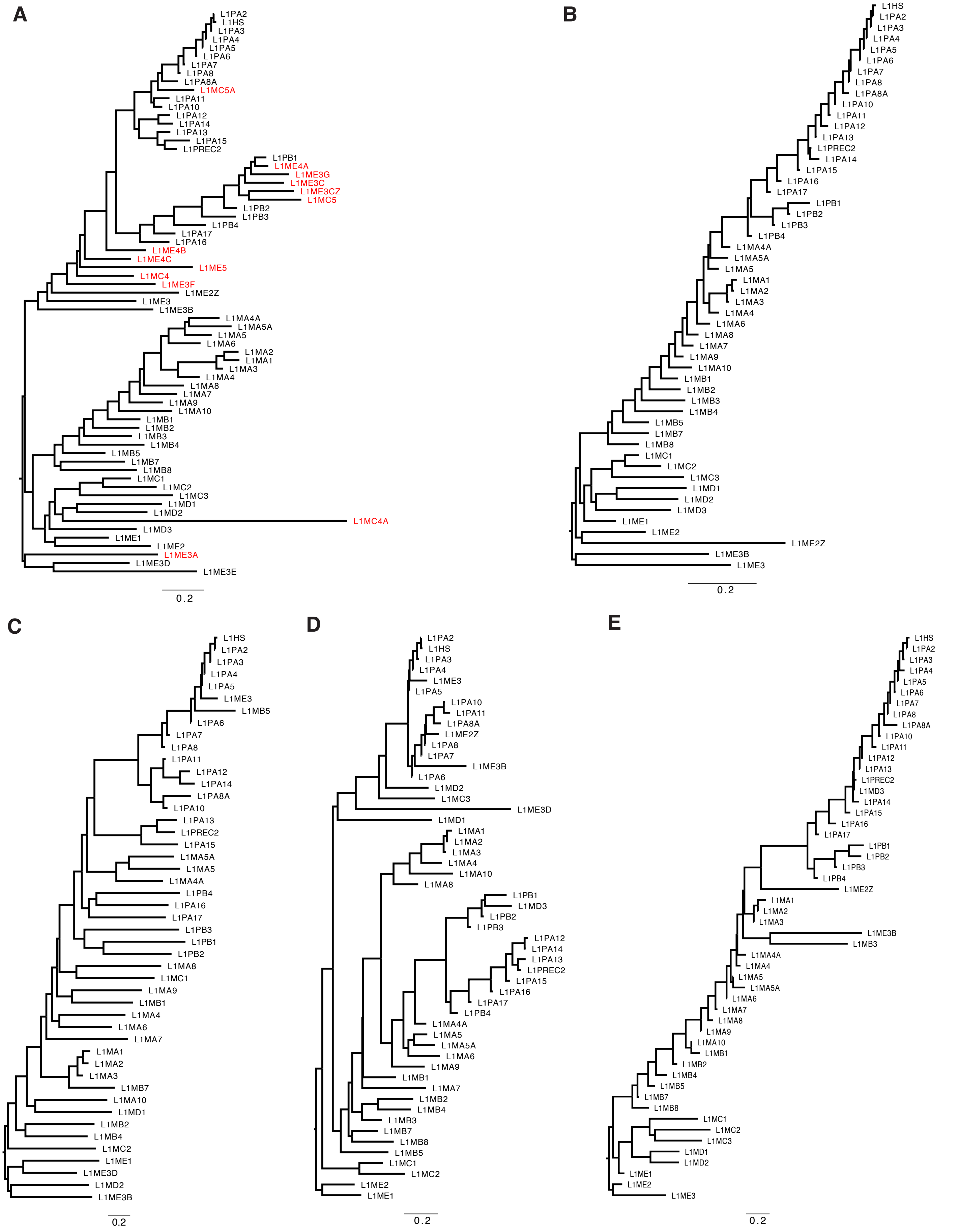

Figure S15. Composite Sequence component trees.
A. Phylogenetic tree of all 67 final Composite Sequences, produced using the same method as Figure 4. Subfamilies that were removed due to irreconcilable difference from the expected tree topology are in red text.
B-E. Phylogenetic trees were produced using different components of the composite sequences – **B.** ORF2, **C.** 5'UTR, **D.** ORF1, and **E.** 3'UTR.

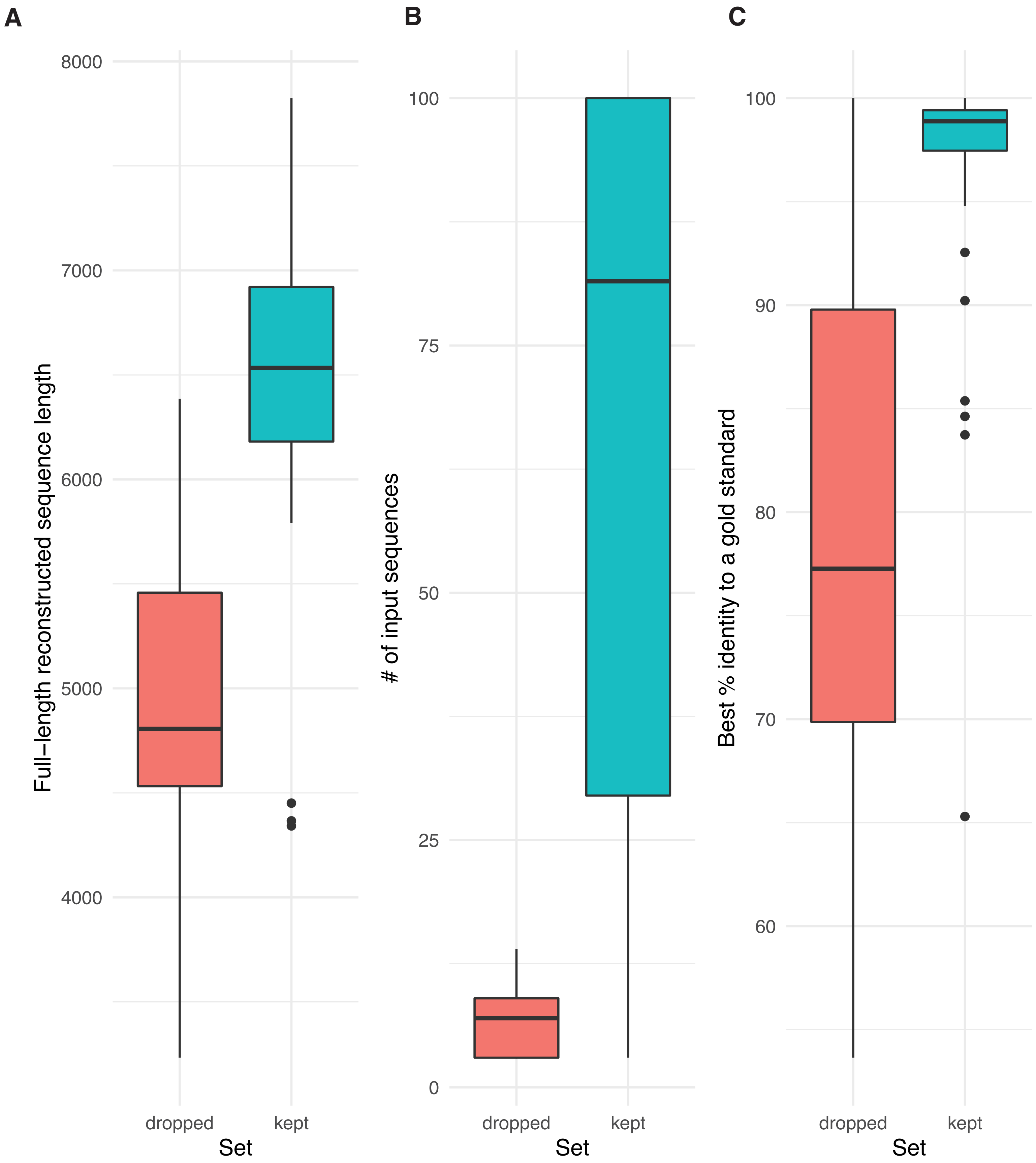

Figure S16. Comparison of properties of the 13 Composite Sequences that were removed from further analysis (red) and the remaining 54 sequences.
A. Lengths of the initial full-length reconstructed sequences.
B. Number of sequences input into the initial reconstruction.
C. Maximum % identity to any of a subfamily's gold standard sequences.

Figure S15

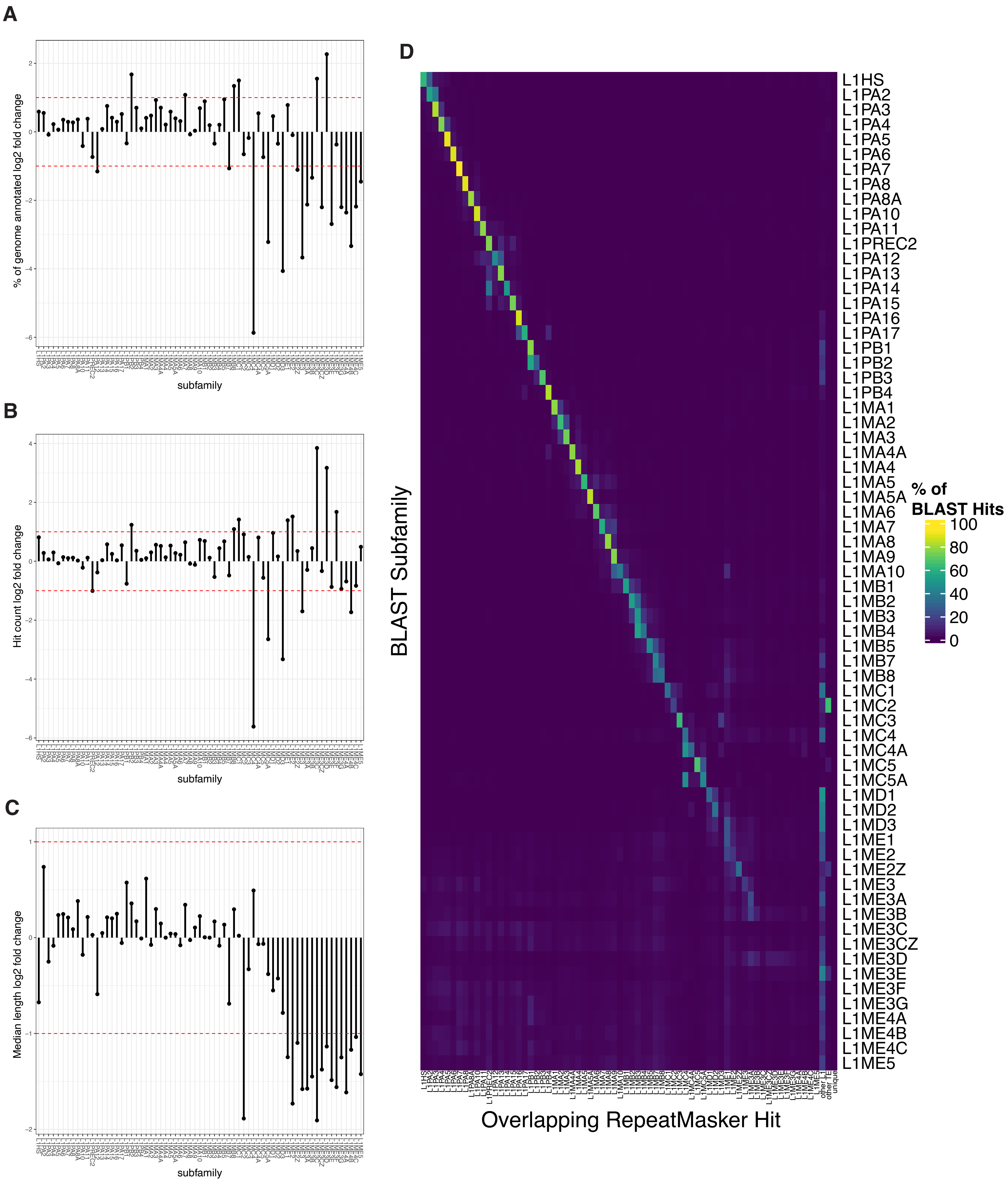
A
% of genome annotated log2 fold change
subfamily
B
Hit count log2 fold change
subfamily
C
Median length log2 fold change
subfamily
D
BLAST Subfamily
Overlapping RepeatMasker Hit
% of BLAST Hits
100
80
60
40
20
0
L1HS
L1PA2
L1PA3
L1PA4
L1PA5
L1PA6
L1PA7
L1PA8
L1PA8A
L1PA10
L1PA11
L1PREC2
L1PA12
L1PA13
L1PA14
L1PA15
L1PA16
L1PA17
L1PB1
L1PB2
L1PB3
L1PB4
L1MA1
L1MA2
L1MA3
L1MA4A
L1MA4
L1MA5
L1MA5A
L1MA6
L1MA7
L1MA8
L1MA9
L1MA10
L1MB1
L1MB2
L1MB3
L1MB4
L1MB5
L1MB7
L1MB8
L1MC1
L1MC2
L1MC3
L1MC4
L1MC4A
L1MC5
L1MC5A
L1MD1
L1MD2
L1MD3
L1ME1
L1ME2
L1ME2Z
L1ME3
L1ME3A
L1ME3B
L1ME3C
L1ME3CZ
L1ME3D
L1ME3E
L1ME3F
L1ME3G
L1ME4A
L1ME4B
L1ME4C
L1ME5

Figure S17. Composite Sequences and BLAST annotations of hg38 and comparison to RepeatMasker and Dfam models.

A-C. Log2 fold changes, by subfamily, of the three annotation quality metrics shown in Figure 6 (BLAST annotations/RepeatMasker annotations). Dotted red lines indicate fold changes of 2 and 0.5. **A.** Percent coverage of genome. **B.** Count of individual L1 instances. **C.** Median lengths of L1 instances.

D. Percentage agreement between BLAST-called subfamily assignments and RepeatMasker annotations. In cases where a single BLAST hit overlapped multiple RepeatMasker hits, the element with the greatest overlap with the BLAST hit was selected. BLAST hits that overlapped no RepeatMasker annotation for > 5 bp are considered “unique”.

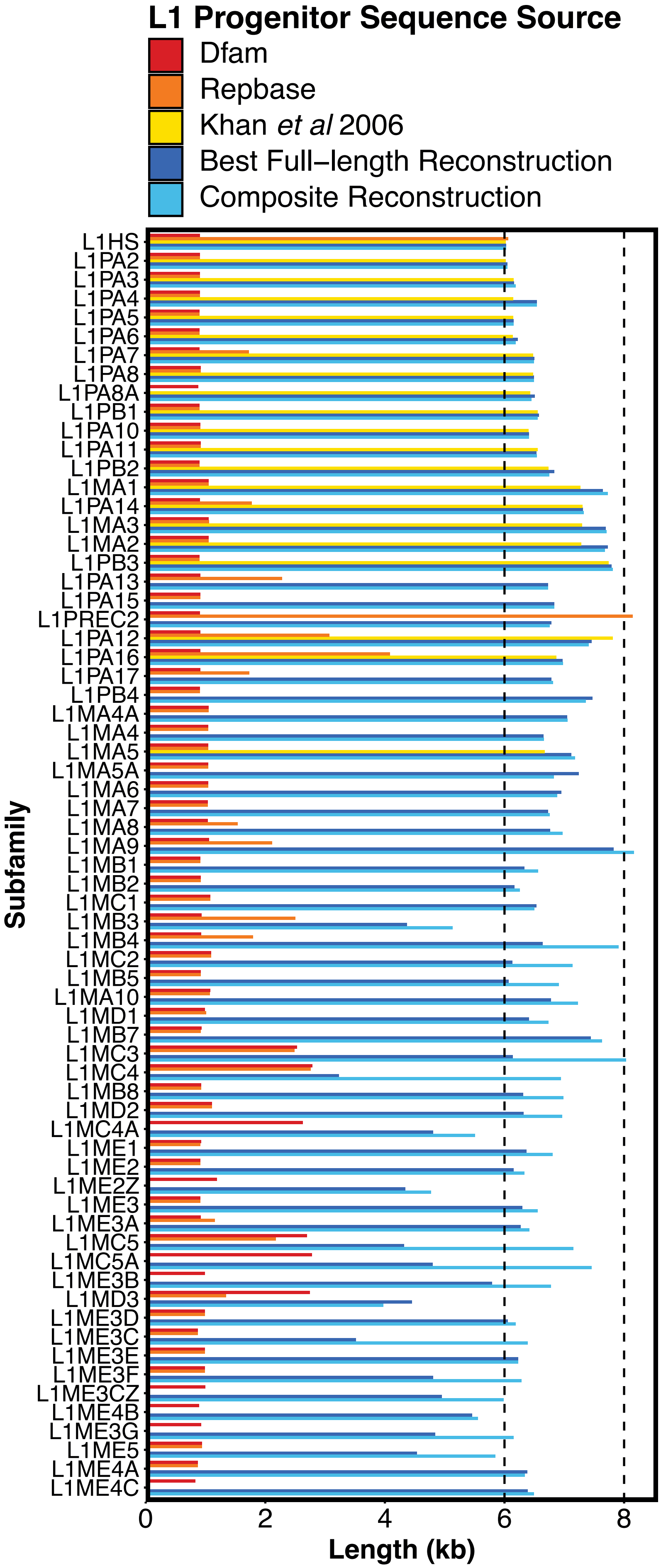

Figure S18. Reconstructed subfamily progenitor sequence lengths relative to existing models.
